# A Batteryless Wireless Microphone Using RF Backscatter

**DOI:** 10.1145/3770673

**Published:** 2025-12-02

**Authors:** QIJUN WANG, CHUNQI QIAN, PEIHAO YAN, SHICHEN ZHANG, HUACHENG ZENG

**Affiliations:** Michigan State University, USA

**Keywords:** Batteryless microphone, backscatter communications, energy-efficient data transmission, radio frequency tags

## Abstract

Wireless microphones are essential tools in business, education, entertainment, and other domains. However, most existing designs rely on batteries, leading to the inconvenience of frequent recharging and the risk of unexpected power failure during use. In this paper, we present TagMic, a battery-free wireless microphone enabled by a novel radio frequency (RF) backscatter technology. TagMic is built on two key innovations. *(i) Parametric backscatter tag design:* This design enables the RF tag to operate at separate excitation and reflection frequencies, fundamentally mitigating the self-interference problem inherent in conventional RFID systems. Unlike harmonic backscatter approaches, it also requires a significantly lower activation voltage, resulting in a longer communication range. *(ii) Voice modulation via RF coupling:* A passive piezoelectric sensor is integrated with the RF tag through RF coupling to enable analog-domain frequency modulation (FM), directly encoding voice signals onto the backscattered signal. This eliminates the need for digital signal processing, allowing for truly continuous voice streaming. We have built a prototype of TagMic and evaluated it under realistic conditions. Extensive experiments demonstrate its effectiveness in achieving battery-free, continuous, and seamless wireless voice streaming in realistic applications.

## 1 INTRODUCTION

Wireless microphones play a crucial role in diverse daily-life applications, including entertainment, education, business, and healthcare [4, 15, 16, 30, 36]. They eliminate physical constraints, enabling users to perform, present, or interact with greater freedom and efficiency. This makes them indispensable tools in both professional and everyday environments. However, most existing wireless microphones rely on batteries for operations [2, 10, 12, 19, 33]. This reliance creates an inconvenience for users, requiring frequent battery recharging or replacement to maintain their seamless voice communications. Moreover, the reliance on batteries introduces the risk of unexpected power failures, which can disrupt critical tasks or performances. These limitations underscore the need for battery-free solutions to enhance user experience and reliability.

RF backscatter has been studied for batteryless microphone applications using both analog and digital modulations. Digital backscatter devices, such as Wireless Identification and Sensing Platform (WISP) [17] and MultiScatter [14], first digitize the voice signal and modulate it onto RF waves for communications. Their approaches are actually not limited to voice communications but also applicable to other sensing signals and modalities. In contrast, analog backscatter devices, such as RF Bandaid [26], MARS [1], Battery-Free Phone [34], aim to reduce or completely remove the need for power-hungry digital circuits and rely on analog circuits for voice sensing and communications. RF Bandaid [26] eliminates digital circuitry by mapping sensor output directly to frequency modulation (FM), enabling ultra-low-power wearable sensing. MARS [1] further reduces power consumption to below 1 μW for speech transmission, powered by ambient energy sources. Battery-Free Phone [34] achieves analog voice communication by modulating a JFET’s impedance with microphone signals, bypassing ADCs and operating on harvested RF and light. It presents a landmark analog backscatter design that enables real-time speech communication while consuming only a few microwatts of power.

Despite the advancement, most of the above RF backscatter approaches rely on the accumulation of harvested energy over a certain period of time to power the RF tag circuits, resulting in a “low-duty-cycle” operation. To date, little progress has been made in the design of viable solutions that can support *continuous, seamless, and reliable* voice streaming from a batteryless device to an anchor device (see §6 and Table 1 for details). To achieve continuous voice streaming, an RF backscatter should address the following two challenges.

- **Challenge #1: Self-Interference**. Most RF backscatter tags, such as RFID, reflect radio signals at the same frequency as their excitation signals. Since the excitation signal is orders of magnitude stronger than the reflected signal, the latter is severely corrupted by self-interference from the excitation signal at the RF reader. This self-interference significantly reduces the sensitivity of the RF reader, resulting in a limited communication range and a high packet error rate (PER) in practical scenarios. While a high PER may be tolerable for time-insensitive RFID applications, it poses a fundamental challenge for voice streaming, which requires a reliable communication channel for uninterrupted data transmission. Therefore, addressing self-interference is critical for enabling effective and robust voice streaming applications.
- **Challenge #2: Voice Signal Modulation**. The power harvested by a typical RF backscatter tag is extremely limited, making it insufficient to support digital voice signal modulation. Analog modulation schemes, which have been explored in prior work [1, 26, 34, 53], appear to be a plausible alternative. However, existing analog modulation techniques are not power-efficient enough for an RF backscatter tag to support continuous voice streaming. This highlights the need for innovations in the design and integration of voice modulation and transmission for batteryless RF tags.

In this paper, we present TagMic, the first-of-its-kind batteryless wireless microphone powered by RF backscatter technology that supports *continuous* voice streaming. TagMic consists of two key components: (i) an RF backscatter tag integrated with a piezoelectric sensor to capture voice signals and (ii) an RF reader responsible for transmitting excitation signals and demodulating voice signals. The RF tag operates by modulating voice signals from the piezoelectric sensor onto the reflective signals. This modulation is achieved by dynamically altering the reflective capacitance properties of the tag, effectively encoding the voice waveform onto the reflected signal. The RF reader then demodulates the backscattered signals to retrieve voice data, enabling *continuous* voice communication without requiring active transmission or an onboard power source on the RF backscatter tag.

TagMic addresses the above two challenges by introducing an innovative backscatter tag design featuring dual resonators: a voltage-sensing resonator (VSR) and a parametric resonator (PR). Both resonators are passive components that leverage non-linear elements (varactor diodes) to produce voltage-tunable resonance frequencies distinct from the excitation signal frequency. The VSR features an ∞-shaped conductor structure with two varactor diodes arranged in a head-to-head configuration. The piezoelectric sensor converts voice sounds into voltage signals, which alter the capacitance of the VSR’s varactor diodes, thereby modulating its resonance frequency. This results in a radio signal whose frequency shift is approximately linearly related to the voice signal. The PR acts as a signal amplifier for the VSR. It consists of a circular conductor structure split by a pair of varactor diodes, also in a head-to-head configuration, and is tightly coupled with the VSR. The resonance frequency shift of the VSR is transduced into an oscillation frequency shift in the PR, producing a frequency-modulated signal that can be wirelessly detected by an anchor RF reader.

This new structure provides three key advantages for the existing backscatter tag. First, its excitation and reflection signals are largely separated in the frequency domain, thereby fundamentally mitigating the self-interference issue in conventional backscatter communications. Second, voice signals are modulated onto the tag’s resonance frequency by altering the varactor diode’s capacitance, eliminating the need for digitalization. This enables continuous voice streaming on a batteryless backscatter tag, addressing the second challenge. Third, the tag achieves frequency modulation, establishing a linear relationship between the tag’s resonance frequency shift and the voice signal amplitude. This simplifies voice recovery at the RF reader.

In addition to the backscatter tag, we have developed a signal processing pipeline for the RF reader to optimize voice recovery. Our design deals with tag imperfections, such as resonance frequency drift and multi-carrier frequency suppression, to ensure reliable voice streaming. Notably, we develop a robust algorithm for carrier frequency offset (CFO) compensation and implement a lightweight deep learning model that leverages inherent voice properties to mitigate nonlinear noise, enhancing voice quality. We have fabricated the proposed backscatter tag and built a prototype of TagMic. Extensive experiments demonstrate that TagMic provides a satisfactory voice performance under various realistic conditions and supports the simultaneous operation of multiple tags.

Table 1 compares TagMic against existing RF backscatter work. The contributions of this work are summarized as follows:

- We design and demonstrate a batteryless wireless microphone with significant potential for continuous voice streaming applications across various fields.
- We propose a dual-resonator RF backscatter tag that modulates voice signals onto its resonance oscillation frequency, enabling reliable far-field detection.
- We demonstrate TagMic in realistic scenarios, with extensive experiments confirming its superior performance, multi-tag accessibility, and practical feasibility.

## 2 OVERVIEW

As explained in §1, self-interference is a grand challenge in the design of backscatter-based solutions for battery-free wireless microphones. If the backscatter reflects radio signals at the same frequency as its excitation signal, it becomes difficult for the RF reader to demodulate the voice signal. This is because the excitation signal generated by the RF reader is orders of magnitude stronger than the backscattered signal from the tag. Completely mitigating the self-interference from the excitation signal is extremely challenging for the demodulation of the backscattered signal. Even after self-interference suppression, the residual interference remains dominant compared to the backscattered signal. One approach to addressing the self-interference issue is to separate the frequencies of the excitation and reflection radio waves. If these two frequencies are sufficiently separated, the RF reader can easily mitigate the self-interference from the excitation signal in the frequency domain when decoding the reflective signals from the tag.

One way to achieve this separation is by utilizing harmonic RF tags [18, 48]. Unlike traditional RF tags, harmonic RF tags exploit the *non-linear* properties of the tag’s circuit to generate *harmonic* signals. When an RF signal is transmitted to the tag, the circuit reacts nonlinearly, generating harmonic frequencies (e.g., doubling or tripling the frequency of the incoming signal). These harmonic signals then propagate back to the reader, carrying the tag’s information bits. The RF reader decodes the information from the harmonic signals, rather than from the fundamental frequency. By eliminating self-interference, the RF reader achieves better signal detection sensitivity, offering several advantages over traditional RF backscatter tags, including higher data transfer rates, lower packet error rates, and greater resilience to environmental noise.

While existing harmonic backscatter systems can mitigate self-interference, they are not well-suited for microphone applications for the following reasons: *First*, harmonic backscatter tags rely on non-linear diodes (or other non-linear elements) working in their nonlinear region to generate harmonic signals. Non-linear diodes, however, have a minimum threshold voltage that must be reached before they exhibit non-linear behavior and generate harmonics. This means that the incident (excitation) signal must be strong enough to bias the diode into its non-linear region. This limits the communication range of harmonic tags. *Second*, and more importantly, since the harmonics are generated by a single device such as a diode or transistor, it is extremely difficult to modulate an analog voice signal onto the harmonic radio wave without digitizing the voice signal. This leaves the second challenge (voice signal modulation, see §1) unaddressed.

In light of the issues with harmonic RF tags, we propose a new backscatter tag design for TagMic, which utilizes dual LC resonators to generate an oscillation signal at a frequency different from its excitation signal. Similar to harmonic tags, our LC dual-resonator tag also leverages the nonlinear properties of varactor diodes to alter the resonance frequency based on the external voice signal. Unlike harmonic tags, our LC tag does not require the varactor diode to be activated in the nonlinear region to generate harmonics. Instead, it utilizes the voltage-tunable capacitance feature of the varactor diode to dynamically change its resonance frequency. More importantly, the voice signal can be directly modulated onto the resonance frequency, achieving frequency modulation without the need for voice signal digitization. We present our design in the following section.

## 3 DESIGN: RF BACKSCATTER TAG

### 3.1 Preliminaries

#### LC Resonator.

An LC resonator is an electrical circuit consisting of an inductor (L) and a capacitor (C) connected in a specific configuration that exhibits resonance at a particular frequency. When an excitation RF signal is applied to the LC resonator, it oscillates at its resonant frequency, which is determined by the values of the inductor and capacitor. The resonant frequency is a function of the inductance (L) and capacitance (C) values, and can be adjusted by modifying these components. This characteristic makes LC resonators ideal for various applications, such as frequency generation and filtering, where the resonator produces a predictable and stable output frequency when energized by an external signal. LC resonators are widely used in communication systems, RF circuits, and sensors due to their simplicity, efficiency, and ability to generate precise frequencies with low power consumption.

#### Voltage Sensing Resonator (VSR).

A VSR is an electrical resonator, typically based on an inductor-capacitor (LC) configuration, specifically designed so that its resonance frequency is responsive to an applied external voltage. This voltage sensitivity is generally achieved by incorporating nonlinear components, most commonly varactor diodes, whose capacitance varies with the applied bias voltage. When a sensing voltage is connected across specific points of the VSR circuit (often via dedicated sensing electrodes linked to virtual grounds), it modulates the capacitance of these varactors. This change in capacitance alters the total equivalent capacitance of the resonator, thereby shifting its characteristic resonance frequency. This principle allows the VSR to effectively transduce a voltage input into a measurable frequency variation, serving as a core element in various passive sensing applications. Under conditions where the sensing voltage is significantly smaller than the varactor’s inherent junction potential, the relationship between the input voltage and the resulting frequency shift is often approximately linear. This property makes the VSR suitable for encoding analog voltage signals—such as those generated by a piezoelectric sensor responding to voice—directly into frequency variations, enabling frequency modulation of a radio signal.

#### Parametric Resonator (PR).

A PR is a resonator that generates and controls oscillations through a nonlinear parametric process, where the resonance frequency is modulated by an external signal (e.g., voice signal). Unlike traditional resonators, which have a fixed resonance frequency determined by their physical components (such as inductance and capacitance in LC circuits), a PR’s frequency is dynamically adjusted by modulating a system parameter, such as capacitance, inductance, or nonlinearity of the circuit. This process allows the resonator to produce oscillations at different frequencies depending on the modulation signal.

In a frequency-modulated PR, the resonance frequency is influenced by an external time-varying signal. This modulation causes the resonator to oscillate at varying frequencies over time, producing frequency-modulated signals. The frequency modulation arises because the resonator’s response is dependent on how the system parameters change, effectively causing the resonant frequency to vary with the modulation signal. This behavior is often achieved using nonlinear components, such as varactors or other types of nonlinear capacitors and inductors, that allow for periodic adjustments in the resonance frequency.

### 3.2 Our Design

#### Tag Structure.

**Fig. 1.**
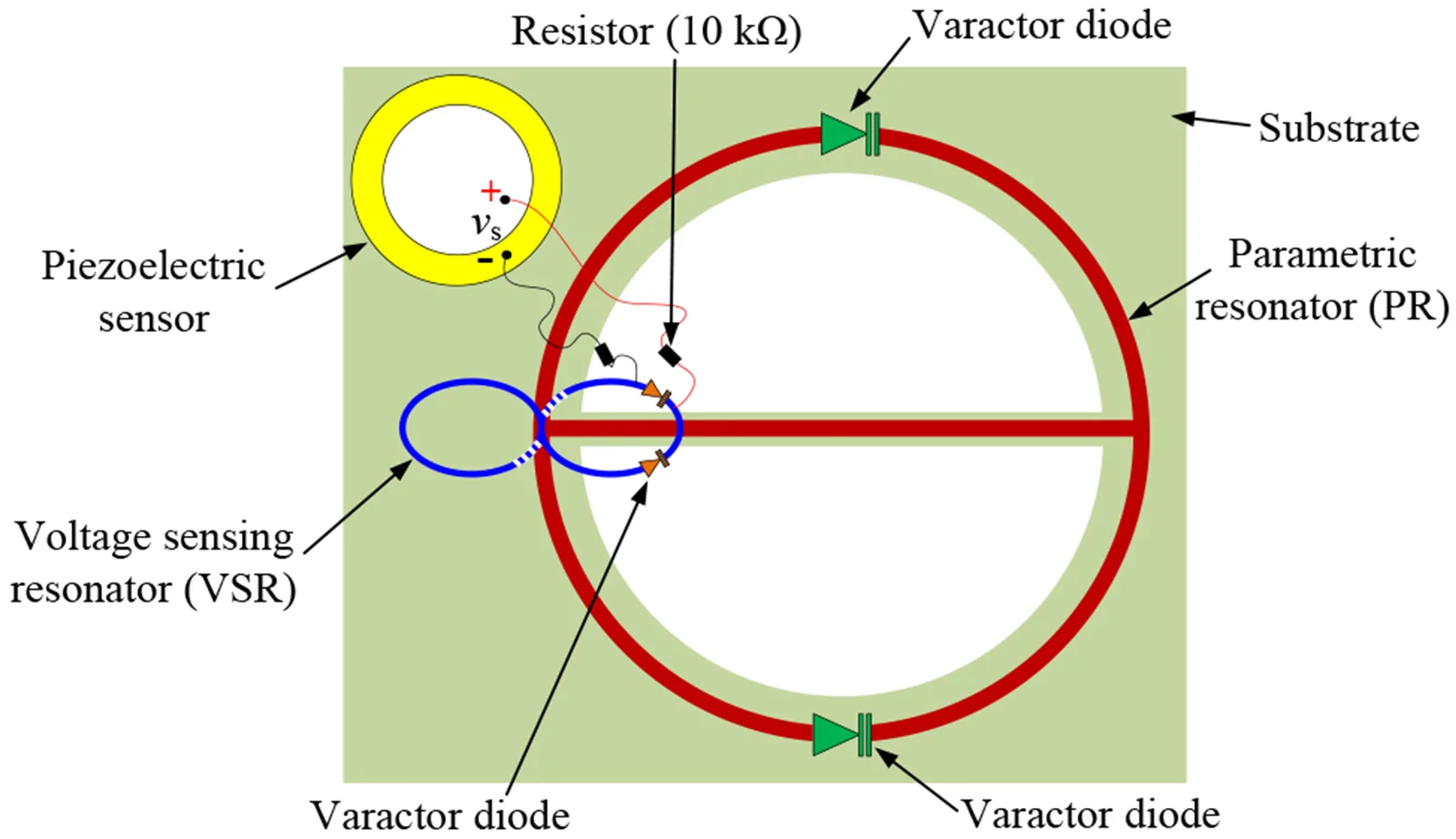
The RF tag includes a voltage sensing resonator (VSR), a parametric resonator (PR), and a piezoelectric sensor (PS). The VSR has a ∞-shaped conductor structure with two head-to-head varactor diodes. The PR has a circular-shaped conductor with two head-to-head varactor diodes as well. The VSR is sitting at the edge of the PR, creating effective coupling with PR for frequency generation.

Fig. 1 shows our design of the RF backscatter tag. It includes a VSR, a PR, and a piezoelectric sensor, all of which are passive devices without the need for a battery. The piezoelectric sensor converts voice sound to a voltage signal, which alters the capacitance of the varactor diodes and therefore changes the resonance frequency of the VSR. The VSR has an ∞-shaped conductor structure made of an enameled copper wire wrapped around two parallel rods. The wire’s two end edges are soldered to two varactor diodes connected in a head-to-head configuration.

**Fig. 2.**
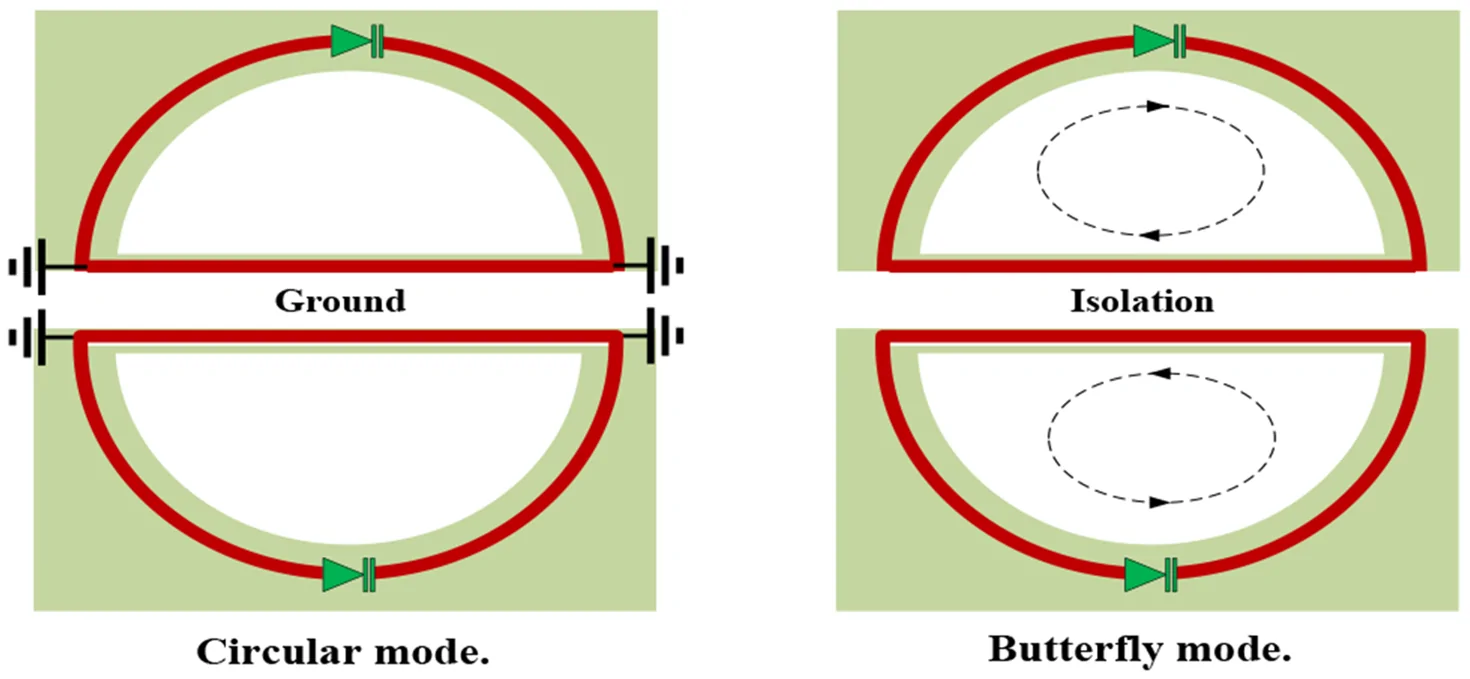
The PR operates in the circular resonance mode (left), where the circuit has zero voltages in the center plane, making the circular mode equivalent to two half-loops sharing the same center ground. The PR operates in the butterfly resonance mode (right), where two separate current flows are confined within their individual meshes, making the butterfly mode equivalent to two half-loops that are electrically isolated in the center plane.

To improve the detectability, we place the VSR across the edge of the PR, which operates as a local signal enhancer. The PR has a circular-shaped conductor pattern split by a pair of varactor diodes connected in a head-to-head configuration, creating a resonance mode with the circular-shaped current flow. The PR also has a continuous center conductor to create a second resonance mode with the butterfly-shaped current flow. Therefore, the PR has two resonance modes: *circular* mode and *butterfly* mode, as shown in Fig. 2. Through repeated signal mixing of these two modes, the net effect is a significant amplification of signals near both resonance frequencies.

Since the VSR overlaps along the edge of the PR, this configuration establishes effective magnetic coupling between the VSR’s resonance and the PR’s circular-mode resonance. Because the loops of the VSR are symmetrically aligned with the horizontal center conductor of the PR, the VSR is decoupled from the PR’s butterfly mode. Consequently, any shift in the VSR’s resonance frequency—caused by voice sound—will be reflected in the PR’s circular-mode resonance frequency.

#### Separation of Excitation and Reflection Frequencies.

When the tag is activated by a wireless excitation signal at the sum frequency of the circular and butterfly mode resonance frequencies of the PR, the varactor diodes in the PR can convert wireless pumping power into sustained current flows at the PR’s resonance modes, thus sustaining continuous circuit oscillation. When acoustic sound hits the piezoelectric sensor, it creates a bias voltage to modulate the VSR’s resonance frequency. Because the VSR couples only to the circular resonance mode of the PR, the resonance frequency shift of the VSR is converted into circular-mode oscillation frequency shift of the PR, creating a frequency-modulated oscillation signal that can be wirelessly detected by a remote RF reader.

Mathematically, denote $f_{s}$ and $f_{b}$ as the oscillation frequencies of the PR operating in circular and butterfly modes, respectively. These two frequencies are determined by the design of the tag parameters, materials, layout, etc. Then, the excitation frequency of the tag should be $f_{ex}=f_{c}+f_{b}$, and the reflection frequency of the tag is $f_{c}$. The excitation and reflection frequencies of the tag are distinct, fundamentally resolving the self-interference problem. The separation of the excitation frequency $f_{ex}$ and reflection frequency $f_{c}$ makes it easy for an RF reader to demodulate the voice signal over a long distance.

### 3.3 Operation Principles

In this part, we explain the operation principles of VSR and PR, and show that the generated oscillation signal is frequency-modulated by voice sound.

#### Voltage-Sensing Resonator.

**Fig. 3.**
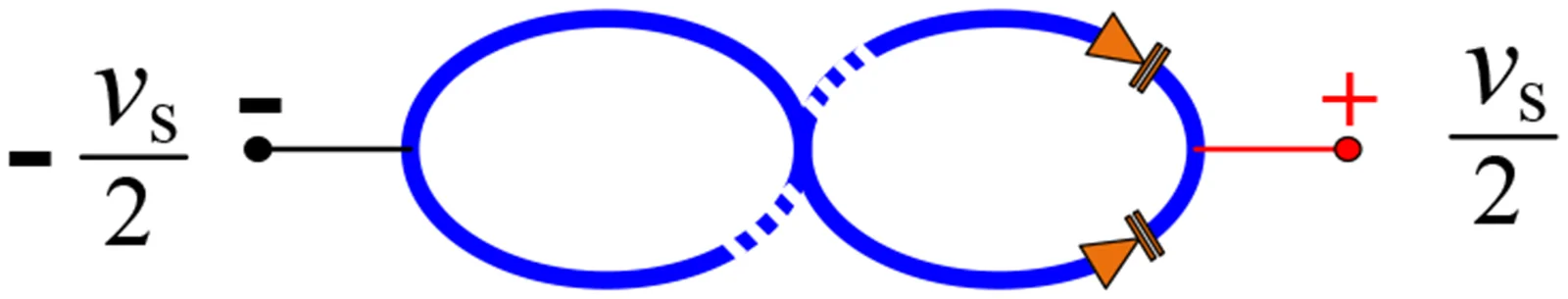
Architectural diagram of VSR.

Fig. 3 illustrates the VSR operating on its own (without PR). Denote $V_{s}$ as the bias voltage generated by the piezoelectric sensor. Denote $f_{1}$ as the resonance frequency of the VSR. When the bias voltage $V_{s}$ is applied to the sensing electrodes, which are connected to the resonator’s virtual grounds, it alters the capacitance of the varactor diodes and consequently shifts the resonance frequency $f_{1}$:

(1) $$f_{1}=\frac{1}{2\pi }\sqrt{\frac{2}{L_{1}C_{1}}}=\frac{1}{2\pi }\sqrt{\frac{2}{L_{1}C_{10}{\left(1-\frac{V_{s}}{\Phi_{1}}\right)}^{-\lambda_{1}}}},$$

where $L_{1}$ and $C_{1}$ are the VSR’s inductance and capacitance, respectively; $C_{10}$ is its capacitance when the bias voltage is 0. $\Phi_{1}$ is the varactor diode’s junction potential. $\lambda_{1}$ is a constant related to the property of the varactor diode.

Consider the case where the sensing voltage $V_{s}$ is much smaller than the diode’s junction potential $\Phi_{1}$. Based on Taylor series approximation, Eq. (1) can be approximated as:

(2) $$f_{1}\approx \frac{1}{2\pi }\sqrt{\frac{2}{L_{1}C_{10}}}\left(1-\frac{\lambda_{1}V_{s}}{\Phi_{1}}\right)\overset{\left(a\right)}{=}f_{10}\left(1-\frac{\lambda_{1}V_{s}}{\Phi_{1}}\right),$$

where Eq. (a) follows our definition that $f_{10}\equiv \frac{1}{2\pi }\sqrt{\frac{2}{L_{1}C_{10}}}$. Conceptually, $f_{10}$ is the VSR’s resonance frequency when the bias voltage is zero.

Eq. (2) reveals that when the sensing voltage $V_{s}$ is much smaller than the varactor diode’s junction potential $\Phi_{1}$, the resonance frequency of the VSR has an approximately linear relationship with the sensing voltage $V_{s}$. This is essentially frequency modulation, with the sensing voltage $V_{s}$ acting as the original signal and $f_{1}$ as the carrier frequency. In our design, we place two large resistors with the VSR’s varactor diode (see Fig. 1), ensuring that $V_{s}$ is much smaller than $\Phi_{1}$.

#### Parametric Resonator.

The PR is a signal enhancer for the VSR to improve the signal emission. Due to circuit symmetry, the PR can be equivalently modeled as two single-frequency resonators, where the effective inductance depends on the resonance mode under consideration. When the PR is powered by an electromotive force oscillating at frequency $f_{p}$, which is wirelessly provided by the RF reader, the junction capacitance of its varactor diodes can be considered modulated by an effective voltage $V_{p}$. By setting the pumping frequency approximately equal to the sum of the resonance frequencies of the circular resonance mode and the butterfly resonance mode, i.e., $f_{p}\approx f_{c}+f_{b}$, the PR leverages the nonlinear capacitance of its varactor diodes to convert external pumping power into enlarged backscattered signals near its circular-mode resonance frequency.

Denote $f_{c}$ as the frequency of the backscattered signals generated by the PR. Recall that $V_{s}$ is the bias voltage signal generated by the voice sound. Then, by analyzing the oscillation frequencies at VSR and PR as well as their coupling relationship, we have:

(3) $$\frac{\partial f_{c}}{\partial V_{s}}=\left(-\frac{\lambda_{1}f_{10}}{2\Phi_{1}}\right)\left(\frac{\frac{L_{c}}{R_{c}}}{\frac{L_{c}}{R_{c}}+\frac{L_{b}}{R_{b}}}\right)\left(\frac{f_{L}^{3}}{2f_{1}^{3}}\right)\left(1+\frac{\frac{f_{2}^{2}}{f_{1}^{2}}-1+2\left(\frac{f_{1}^{2}}{f_{L}^{2}}-1\right)\left(\frac{f_{2}^{2}}{f_{L}^{2}}-1\right)}{|2\frac{f_{2}^{2}}{f_{L}^{2}}-\frac{f_{1}^{2}}{f_{1}^{2}}-1|}\right),$$

where $f_{1}$ and $f_{2}$ are the stand-alone resonance frequencies of VSR and PR, respectively. $f_{L}$ is the lower resonance frequency of the coupled VSR and PR resonators. $R_{c}$ and $L_{c}$ are the effective resistance and inductance of the PR in its circular mode. $R_{b}$ and $L_{b}$ are the effective resistance and inductance of the PR in its butterfly mode.

Evidently, Eq. (3) indicates that the oscillation frequency $f_{c}$ of PR, which is used by the RF reader to demodulate the voice signal, is linearly modulated by the bias voltage $V_{s}$ provided by the piezoelectric sensor. This confirms the frequency modulation property of the proposed tag.

### 3.4 Multi-Carrier Frequency Modulation (FM)

#### Single-Carrier FM.

Ideally, the RF tag modulates the voice signal using frequency modulation, establishing a linear relationship between the voltage from the voice sensor and the instantaneous frequency of the radio signal. Denote v(t) as the voice signal and $f_{c}$ as the frequency of the radio signal emitted by the RF backscatter tag when there is no bias voltage. Then, the ideal FM signal can be written as:

(4) $$s(t)=\alpha \;\text{exp}\left(2\pi f_{c}t+2\pi \Delta \int_{0}^{t}v(\tau )d\tau \right),$$

where α is the signal amplitude, $f_{\Delta }$ is the peak frequency deviation, which controls the extent of frequency shift due to the modulation.

#### Multi-Carrier FM.

However, the imperfections of the electronic devices, such as varactors and other components in the backscatter tag, introduce multiple center frequencies in the backscattered radio signal. The reflection signal from the RF tag can be modeled as:

(5) $$s(t)=\sum_{k=1}^{K}\alpha_{k}\;\text{exp}\left(2\pi f_{c_{k}}t+2\pi \Delta_{k}\int_{0}^{t}v(\tau )d\tau \right)+w,$$

Where $f_{c_{k}}$ is the kth carrier’s center frequency and $\alpha_{k}$ is its amplitude, $\Delta_{k}$ is the frequency deviation sensitivity, and w is the effect of modeling noise and other imperfections of the backscatter tag.

#### Experimental Observations.

**Fig. 4.**
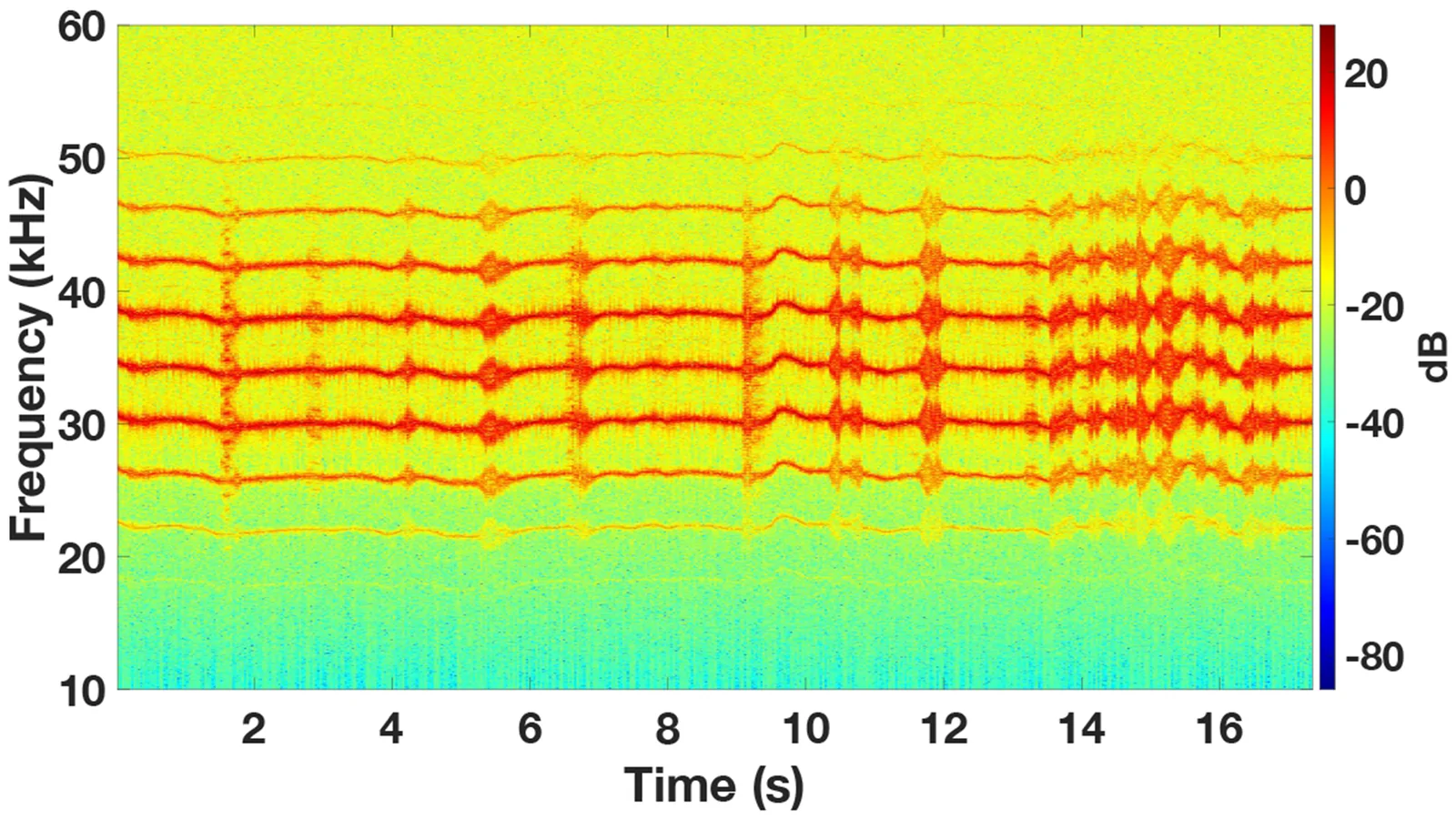
Spectrogram of the RF tag’s reflection signal. The voice signal is frequency-modulated on multiple carriers.

Fig. 4 presents a sample of the measured reflection signals from the RF tag when a voice signal is present. The spectrogram shows approximately six carriers, each representing a copy of a frequency-modulated voice signal. The frequency gaps between these FM carriers are identical. Each carrier can be independently decoded to recover the voice signal. Given that the carriers exhibit varying amplitudes and power levels, different strategies can be employed to leverage spectrum diversity. For instance, the carrier with the largest amplitude can be selected for voice recovery, or all six carriers can be decoded and combined using maximum-ratio combining (MRC) to optimize voice quality.

## 4 RF READER: VOICE RECOVERY

In this section, we present our design for the RF reader to demodulate the backscattered radio signals from the RF tag for voice recovery.

### 4.1 Challenges and Approaches

In designing a voice signal recovery system, we face two significant challenges. The first challenge lies in the carrier frequency offset (CFO) estimation and compensation. The center frequency of the radio signal generated by the backscatter tag is not stable. It fluctuates due to factors such as the tag’s orientation, the movement of surrounding objects, and the frequency drift of the excitation signal. Since FM is sensitive to CFO, this drift degrades the quality of the recovered voice signal at the RF reader. To mitigate this issue, we have developed a CFO estimation algorithm that leverages the signal profile signature to estimate the center frequency offset in real-time. This estimated offset is then compensated for the baseband signal to ensure accurate voice recovery.

The second challenge stems from the inherent nonlinear characteristics of the fabricated RF backscatter tag. While the tag is designed for ideal frequency modulation, its behavior often diverges in practice due to the imperfections caused by electronic components and PCB, rendering the theoretical model inaccurate. Furthermore, the weak backscattered signal recovered at the RF reader is susceptible to multipath propagation effects and residual CFO distortions. These imperfections appear to be non-stationary and complex. Accurately modeling these imperfections through analytical means or addressing them using traditional filtering techniques is extremely difficult. To address this challenge, we propose a learning-based approach that employs a neural network. This data-driven strategy is adept at learning the complex, potentially non-linear mappings between the demodulated, noisy signal and the clean voice counterpart, implicitly capturing and compensating for the system imperfections without requiring explicit mathematical models, thereby enabling robust voice signal recovery and enhancement.

### 4.2 Signal Processing Pipeline

#### Overview.

**Fig. 5.**
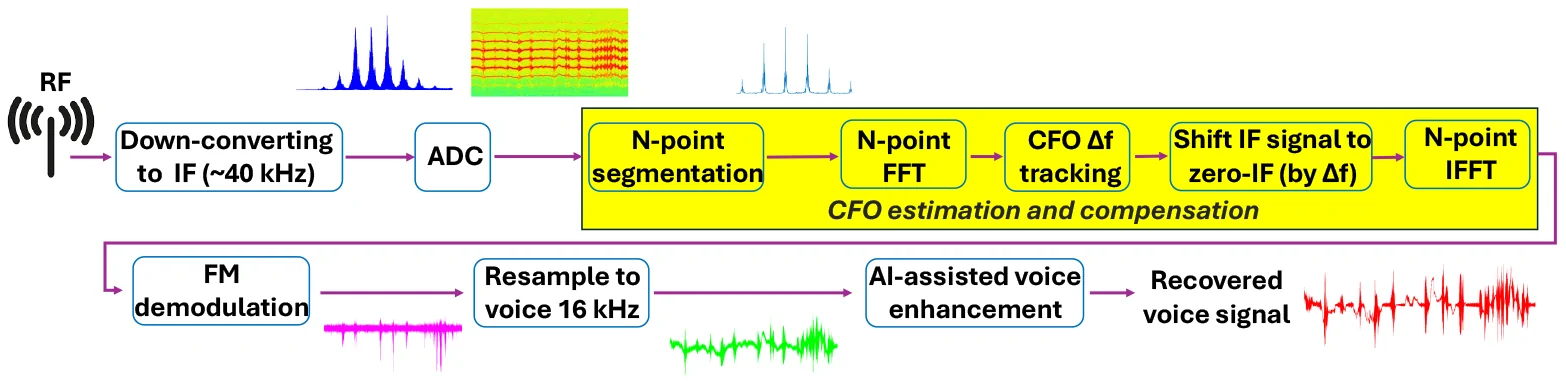
Signal processing pipeline.

Combining the above approaches, we propose a real-time signal processing pipeline, as illustrated in Fig. 5, for the RF reader to recover voice signals. At the RF reader, the radio signal is first down-converted to an intermediate frequency (IF) (e.g., 40 kHz) and then sampled for digital signal processing. The reason why we convert the radio signal to IF rather than baseband (zero-IF) is twofold. First, voice signals contain rich low-frequency components (typically below 100 Hz), and the RF front end often suffers from disturbances near the DC component. Therefore, using IF will avoid interference at low frequencies. Second, the radio frequency suffers from CFO. Since the subsequent process needs to compensate the CFO, using IF will not incur an additional processing burden.

The digitalized signal samples are then processed by a sequence of blocks for CFO compensation (marked yellow in Fig. 5). In this process, the CFO is tracked over time and compensated for individual segmented signal frames. Furthermore, one carrier frequency is selected for frequency demodulation, while others are mitigated in the frequency domain for simplicity. After CFO compensation, the signal is FM demodulated and resampled to the voice sample rate. A lightweight learning-based model has been developed and trained to enhance the voice quality by tackling imperfections of the system including device nonlinearity and unexpected spectral spurs.

#### CFO Compensation.

**Fig. 6.**
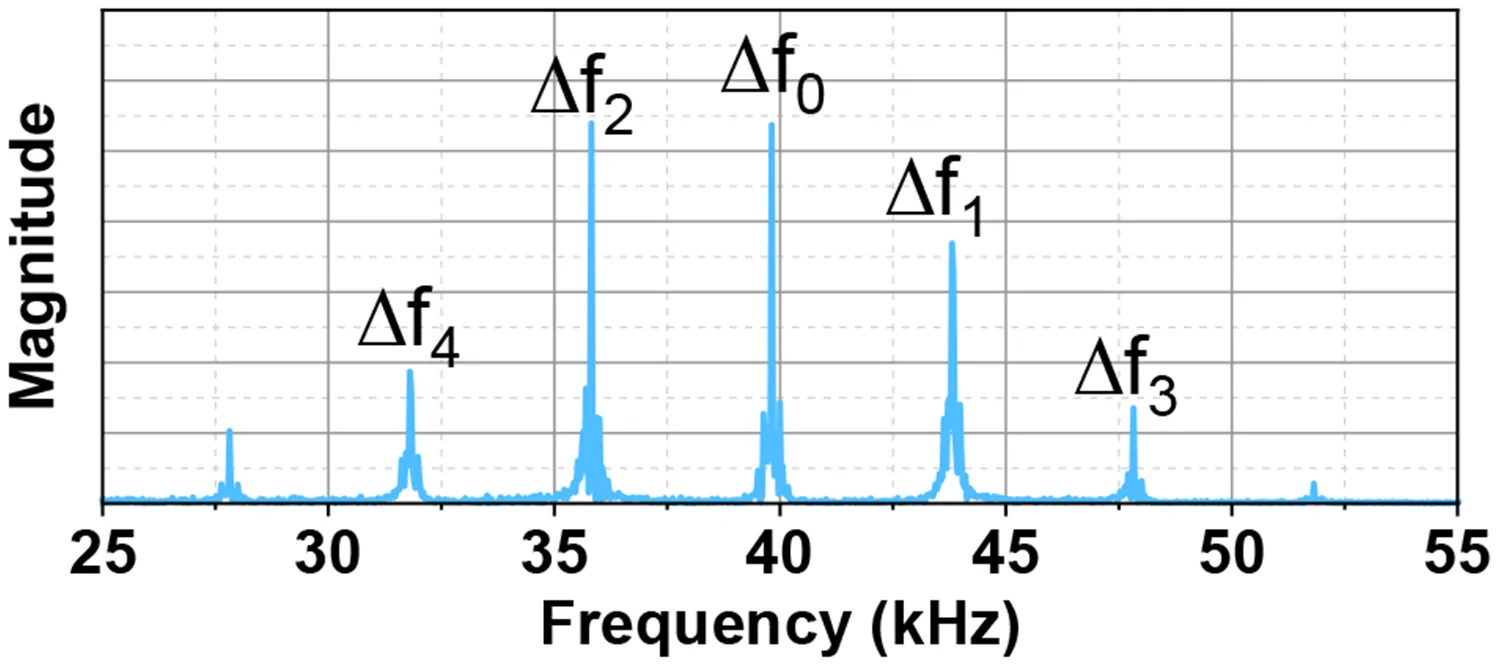
Illustration of the multiple frequency-modulated carriers and their corresponding CFOs.

Our CFO estimation and compensation are performed in the frequency domain. As shown in Fig. 5 (with those blocks highlighted yellow), the signal stream is first segmented and then processed with an FFT operation, converting the signal from the time domain to the frequency domain for CFO estimation, tracking, and compensation. The time duration of each segment is an empirical parameter (e.g., 50 ms). Fig. 6 shows an example of one segment, which has multiple frequency peaks corresponding to multiple carrier frequencies from the RF tag. Among these carrier frequencies, we choose one (e.g., the one with the highest amplitude) for voice signal demodulation. Denote $\Delta f_{k}$ as the center frequency of the kth carrier. We intend to use the one with $\Delta f_{0}$ for voice demodulation. Since voice has rich low-frequency components, $\Delta f_{0}$ must be accurately estimated for CFO compensation.

**Fig. 7.**
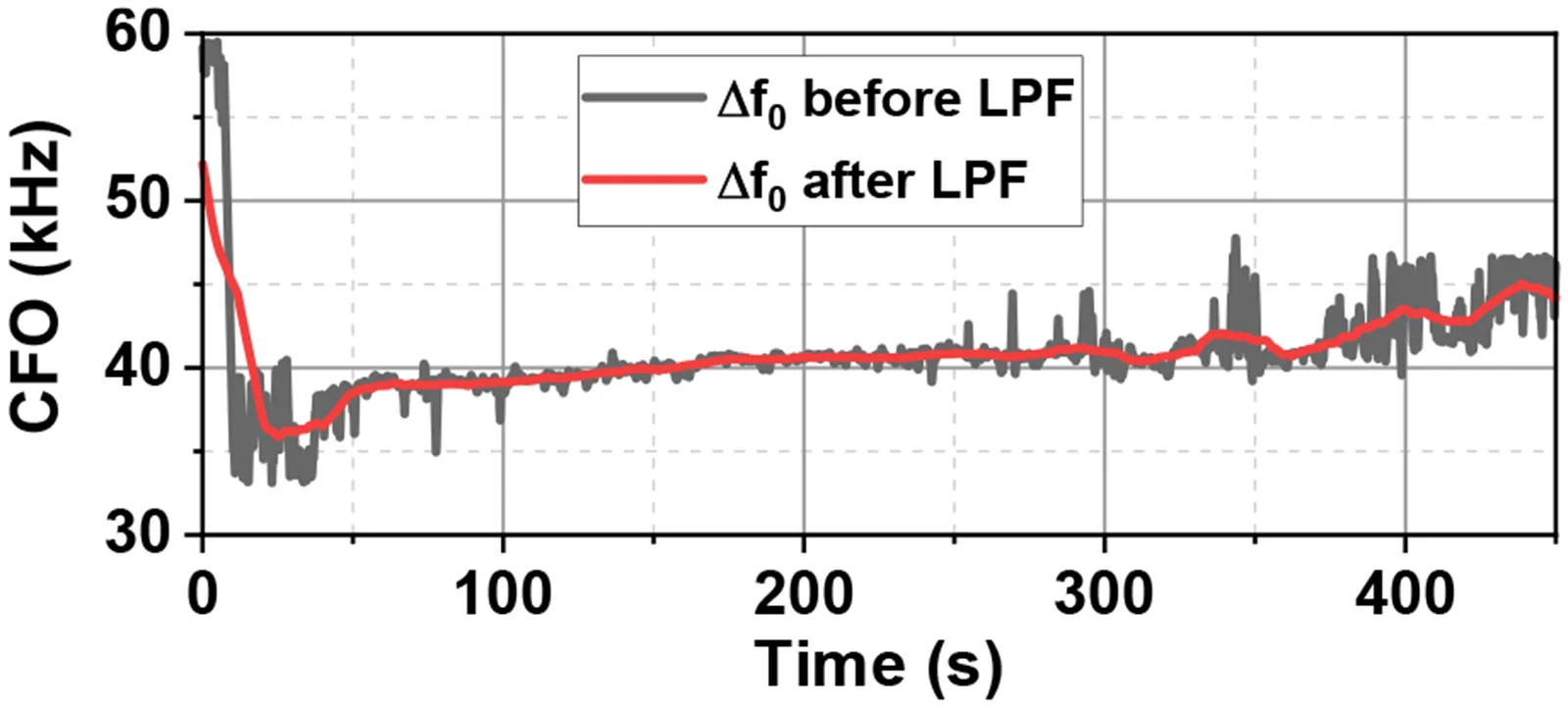
Measured CFO before and after an LPF.

To enhance the CFO estimation accuracy, we employ two strategies: *(i) Averaging over carriers*. Experimental measurement shows that the frequency gap of those carriers is identical. Based on this observation, we first estimate all five CFOs (i.e., $\Delta f_{0},\Delta f_{1},\ldots ,\Delta f_{4}$) using their peak positions, and then calculate $\Delta f_{0}=1∕5\sum_{k=0}^{4}\Delta {\hat{f}}_{k}$, where $\Delta {\hat{f}}_{k}$ is the measured CFO of carrier k. *(ii) Averaging over time*. The carrier frequency drift is a slow, stationary process. Therefore, we employ an LPF to enhance the estimate of CFO over segments. Denote $\Delta f_{0}(t)$ as the CFO of the selected carrier in segment t. Fig. 7 exhibits an example of the measured $\Delta f_{0}(t)$ before and after an LPF with 2 kHz bandwidth.

**Fig. 8.**
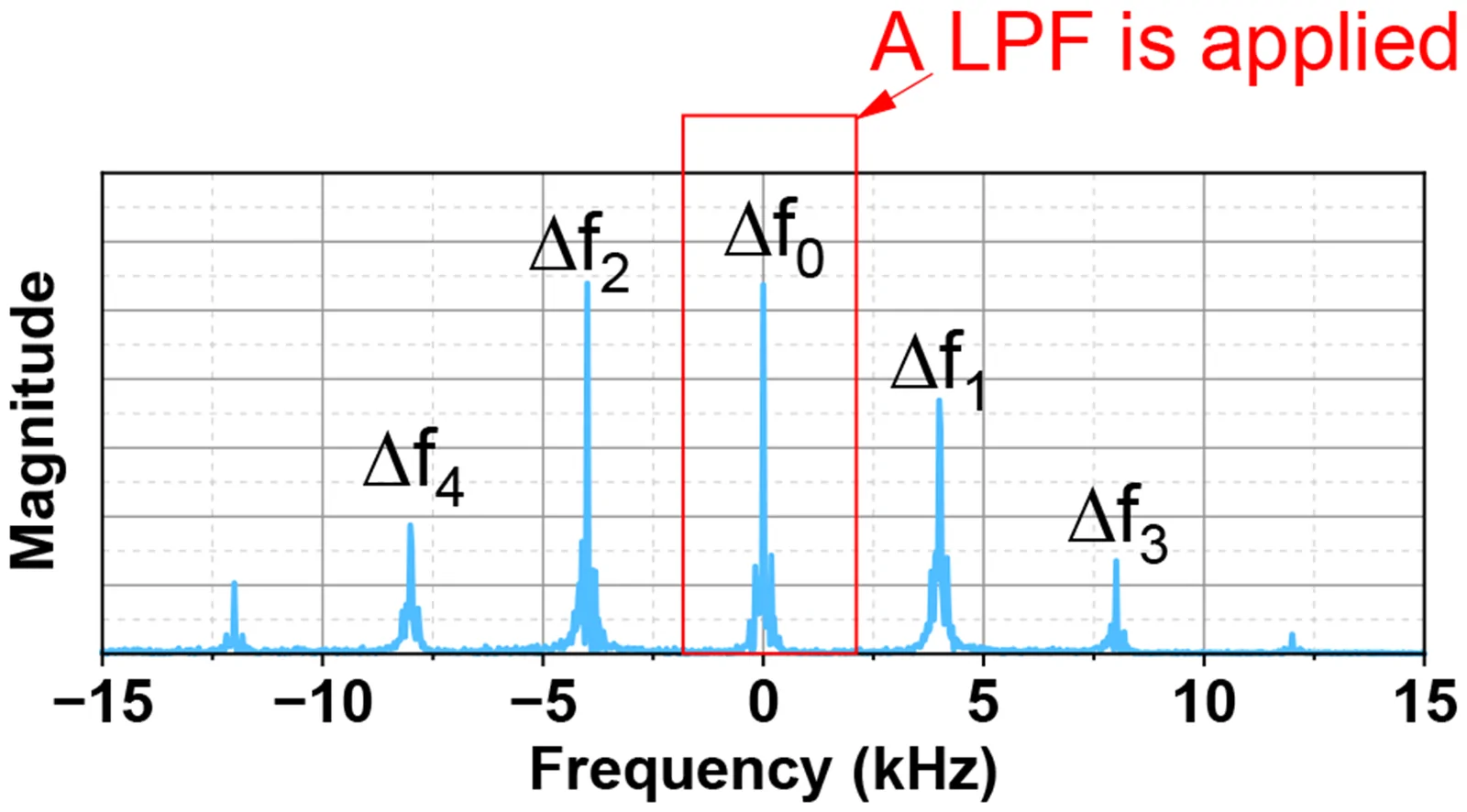
Shift voice signal from IF to baseband and mitigate unintended carriers.

Based on the resultant $\Delta f_{0}(t)$, we shift the center frequency of the intended carrier to zero, generating the zero-IF signal. Then, another LPF is applied to the zero-IF signal to remove other carriers. Fig. 8 shows these operations.

#### Frequency Demodulation.

We use the *phase differentiation* approach to demodulate the zero-IF FM signal. Specifically, referring to Eq. (4), the zero-IF signal after the LPF can be written as: $r(t)=\beta \;\text{exp}\left(2\pi \Delta \int_{0}^{t}v(\tau )d\tau \right)$. Then, the extracted phase can be expressed as: $∠r(t)=2\pi \Delta \int_{0}^{t}v(\tau )d\tau$. Then, we have: $∠r(t_{2})-∠r(t_{1})=2\pi \Delta \int_{t_{1}}^{t_{2}}v(\tau )d\tau \approx 2\pi \Delta v(t_{1})(t_{2}-t_{1})$, provided that $\Delta =t_{2}-t_{1}$ is small enough. Therefore, the voice signal can be estimated by

(6) $$v(t)=\frac{f_{s}}{2\pi \Delta }\left[∠r(t_{2})-∠r(t_{1})\right].$$

where $f_{s}$ is the sampling rate of r(t).

### 4.3 AI-Assisted Voice Enhancement

The resampled voice signal is already audible and intelligible, but sometimes with low-frequency and spurious noise. To enhance the voice quality, we employ a lightweight AI model that leverages the inherent voice properties for noise suppression. We chose an AI-based approach because the distortions in TagMic are nonlinear and time-varying, arising from hardware imperfections and multipath effects. Such distortions are difficult to capture with fixed-parameter filters, whereas a neural network can directly learn the mapping from noisy to clean signals. While the literature has a large body of work in this field, we follow the approaches in [6, 31, 46, 47] to create a solution that operates directly on raw waveforms. This approach does not need to convert the voice signal between time and frequency domains, and thus has a relatively low computation cost.

**Fig. 9.**
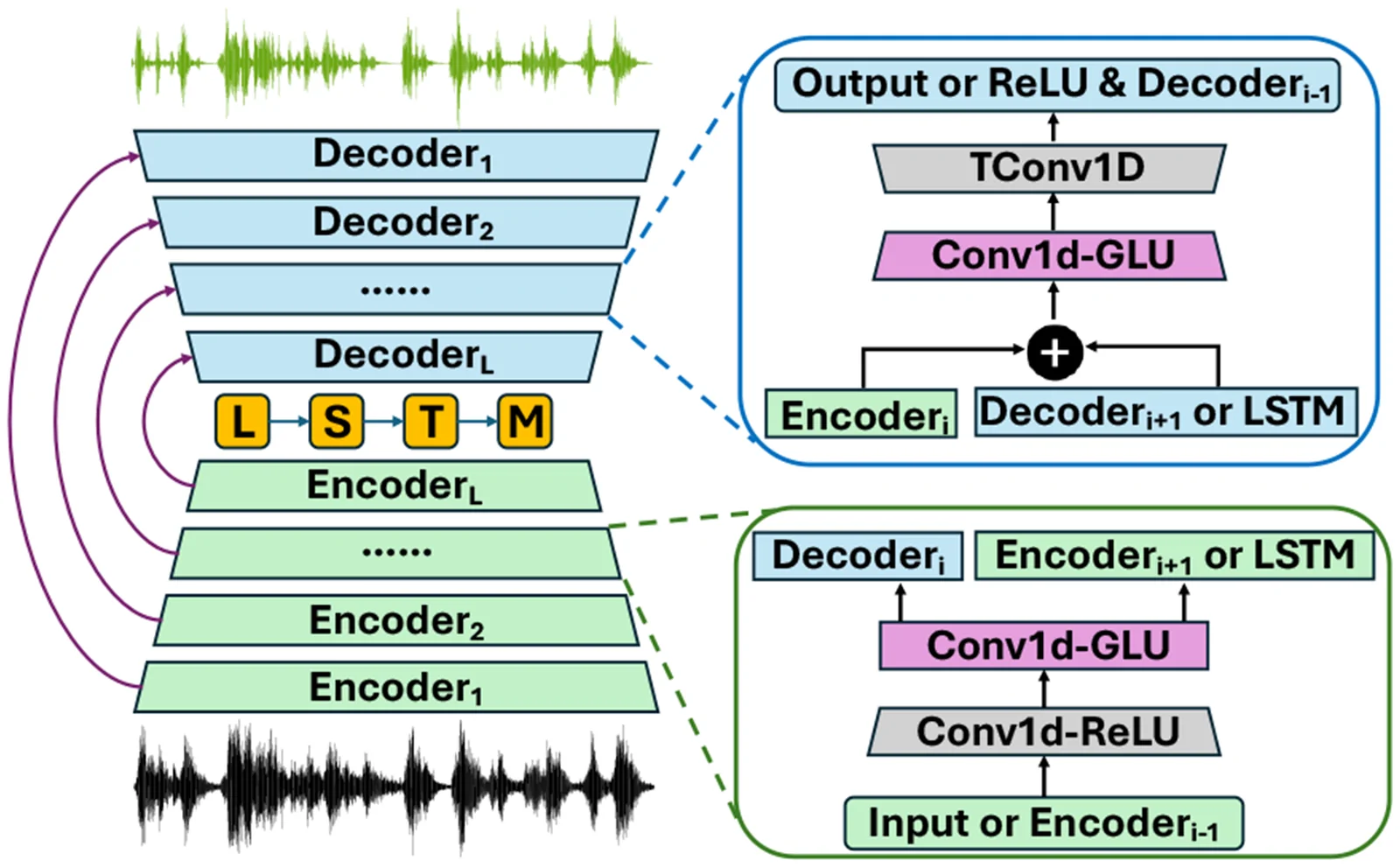
A deep learning model for voice enhancement.

Fig. 9 shows our AI model, which was built based on the DEMUCS [6] architecture. Essentially, it is a causal encoder-decoder framework, augmented with skip connections and LSTM layers for temporal attention. The encoder processes the noisy signal into a latent representation, z=E(x), which is then refined by a sequence model $\hat{z}=R(z)=LSTM(z)+z$. Then, the decoder reconstructs the clean signal, $\hat{y}=D(\hat{z})$. The Skip connections ensure that fine-grained details from the input are preserved across layers, enhancing the output quality.

The training objective is to minimize both waveform-level and spectrogram-domain losses. At the waveform level, the model minimizes the $L_{1}$ loss: ${ℒ}_{w}=\frac{1}{N}{\left\|y-\hat{y}\right\|}_{1}$, which directly measures the difference between the clean signal y and the enhanced signal $\hat{y}$, where N represents the length of the voice signal. To further enhance perceptual quality, a multi-resolution STFT (Short-Time Fourier Transform) loss ${ℒ}_{s}$ is employed. This loss combines spectral convergence and magnitude terms:

(7) $${ℒ}_{s}=\sum_{k=1}^{M}\frac{{\left\|S_{k}(y)-S_{k}(\hat{y})\right\|}_{F}}{{\left\|S_{k}(y)\right\|}_{F}}+\frac{1}{N}{\left\|\log\;S_{k}(y)-\log\;S_{k}(\hat{y})\right\|}_{1},$$

where $S_{k}(\cdot )$ returns the amplitude of a target signal after the k-point STFT operation, ${\left\|\cdot \right\|}_{F}$ and ${\left\|\cdot \right\|}_{1}$ denote Frobenius and $L_{1}$ norms, respectively. M is an empirical parameter representing the number of STFT resolutions. The total loss used to train the model is ${ℒ}_{\text{overall}}={ℒ}_{w}+{ℒ}_{s}$.

**Fig. 10.**
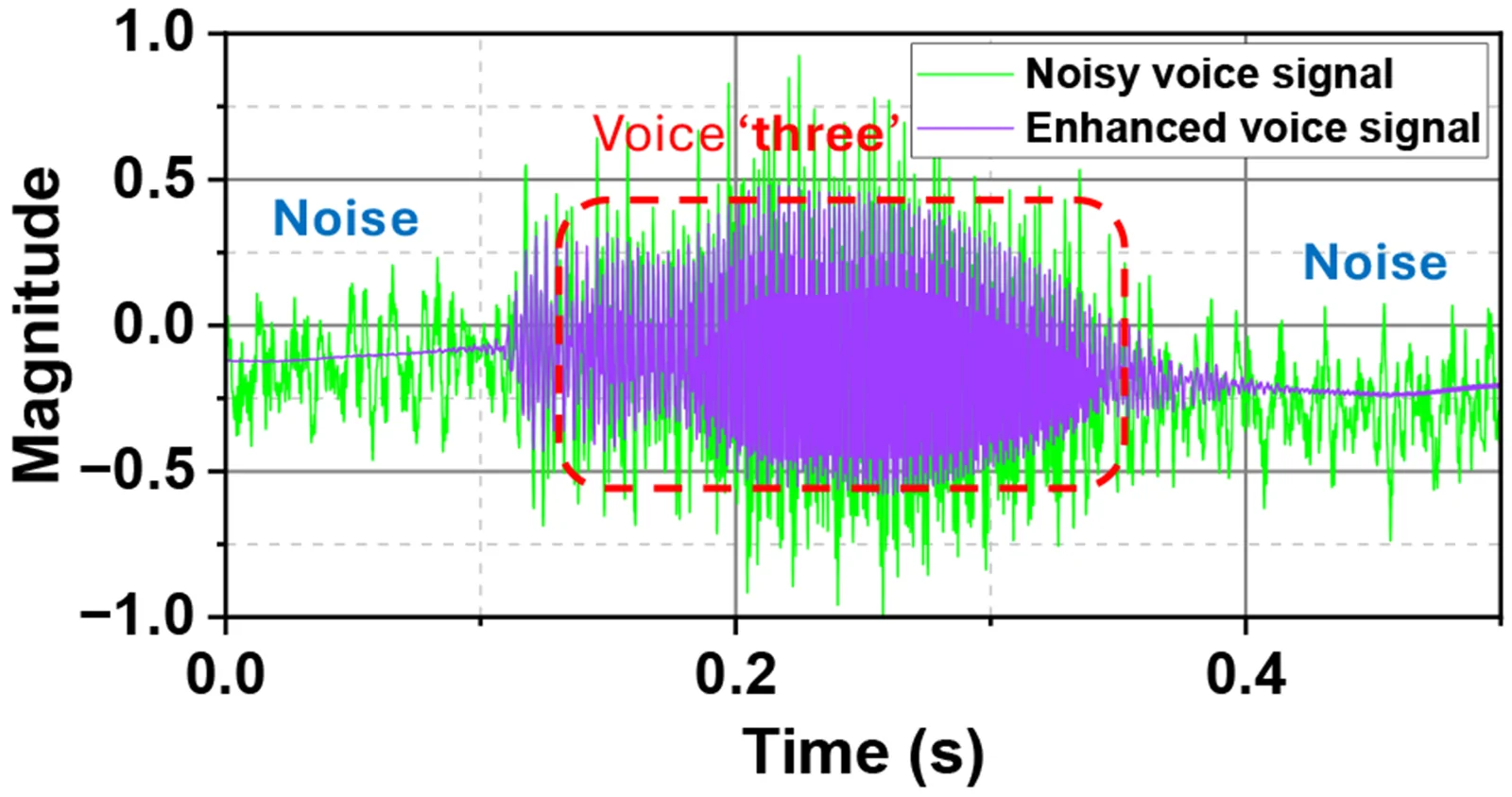
Demodulated voice signal before and after enhancement.

Extensive data from the RF tag, along with its corresponding clean voice data, has been collected to train the AI model. Fig. 10 illustrates an example from the model’s inference stage, showing the input (noisy voice signal) and the output (enhanced voice signal). The results demonstrate that the model effectively suppresses noise and enhances the quality of the voice signal.

## 5 EXPERIMENTAL EVALUATION

In this section, we build a prototype of TagMic and evaluate its performance and robustness in realistic scenarios. Specifically, we aim to seek answers to the following questions.

- **Distance (§**5.4). What is the usual communication distance with an RF reader that has ordinary transmission power (e.g., 1W)?
- **Robustness (§**5.5 and §5.6). How does TagMic perform when the backscatter tag faces different orientations or is in occluded scenarios?
- **Mobility (§**5.7). Does TagMic work when used by walking speakers or in dynamic environments?
- **Multi-User Support (§**5.8). Can an RF reader work with multiple tags at the same time?

### 5.1 Implementation

#### RF Tag.

**Fig. 11.**
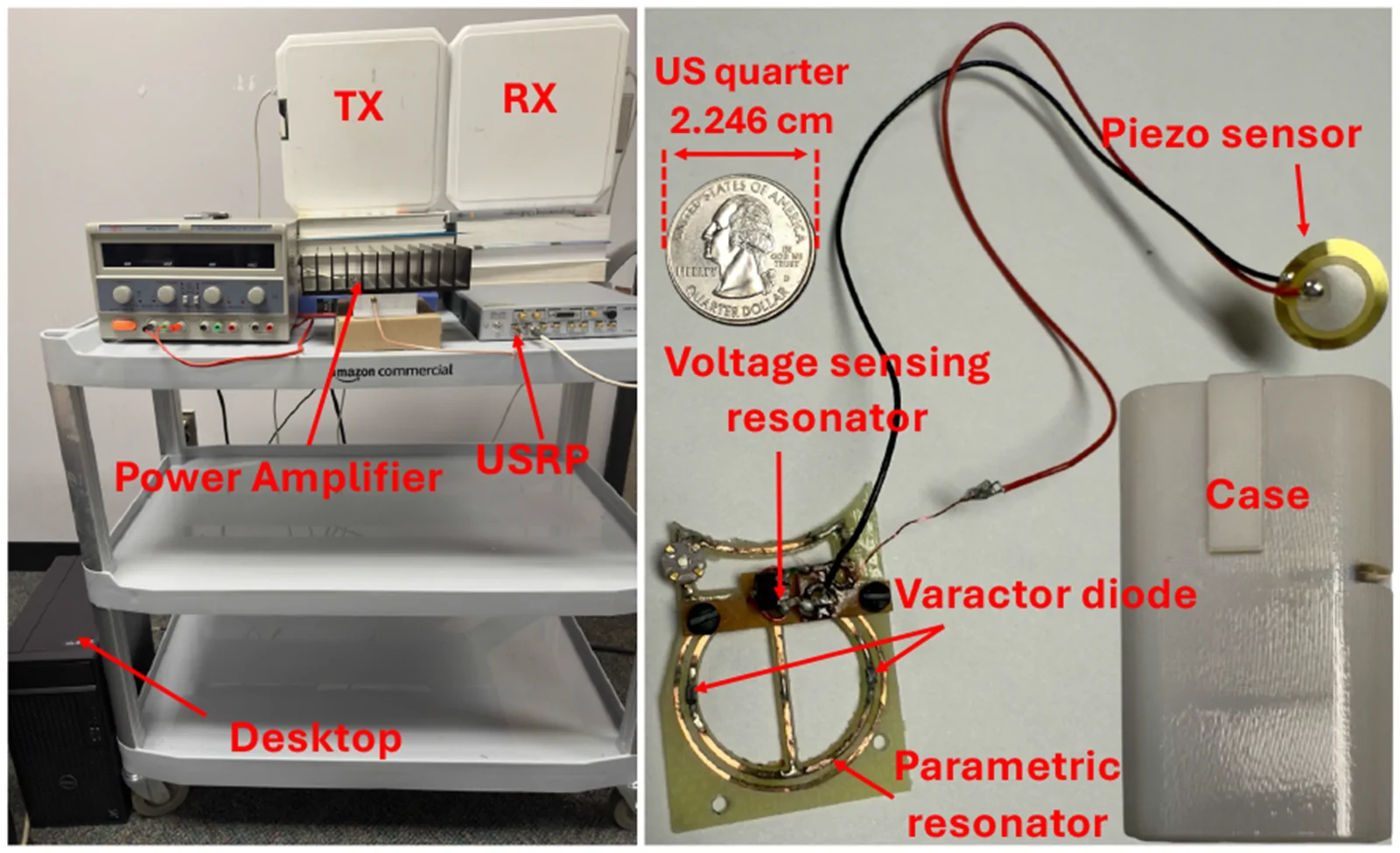
A picture of the RF reader (left) and a picture of the backscatter tag (right).

Fig. 11 shows a picture of our fabricated tag, which consists of a VSR and a PR as well as a piezoelectric sensor. This PR includes a circular inductor with an inner diameter of 13.5 mm and an outer diameter of 14.5 mm etched on the surface of a 0.8-mm G10 board. The upper/right and lower/left half circles have split gaps that were filled by varactor diodes with an equivalent capacitance of 9.1 pF, creating a circular resonance mode. The butterfly resonance mode is created by connecting the two virtual voltage grounds of the circular mode with a horizontal conductor.

The VSR is fabricated by wrapping a 32-G enameled copper wire around two 1.5-mm diameter rods that are separated by 1.8 mm. Five counterclockwise turns in the first rod are followed by a clockwise turn in the second rod, before the wire’s two end terminals are connected to two varactors (3 pF) placed in head-to-head direction. The piezoelectric transducer is connected to the common cathode with a sensing electrode and to the common anode with another sensing electrode. If needed, a half-wave dipole antenna can be wrapped around the edge of the PR’s circular conductor pattern to increase its inductive coupling with the PR. Our prototype uses off-the-shelf components with an estimated material cost of $1.8 per tag. While the current design is handcrafted, we anticipate that the cost can be significantly reduced (to $0.95) with mass production and PCB integration.

#### RF Reader.

Fig. 11 also shows the system setup of the RF reader, which consists of a USRP N310, a power amplifier (PA), two directional antennas, and a PC. The frequency of the excitation signal is 915 MHz, and its transmission power is about 30 dBm. When powered by the excitation signal, the backscatter tag generates reflective signals at about 515 MHz. The entire signal processing pipeline is implemented using GNU Radio on the PC for real-time voice signal demodulation.

To train the AI model in the signal processing pipeline, we collected data in two scenarios. *(i) Human Voice:* Volunteers have TagMic attached to the front of their clothing. Ground-truth voice data was recorded using a smartphone, while the demodulated backscattered signals from TagMic were captured by the RF reader. *(ii) Audio/Video Playback:* To reduce human effort, a smartphone playing YouTube videos was used to collect recovered voice data from the RF reader alongside the corresponding ground-truth voice recordings.

For both methods, data were collected across varying tag distances (50 cm to 550 cm in 50 cm increments) and tag orientations (0° to 180° in 30° increments) to ensure robustness. For each condition, 10–15 minutes of voice data were recorded. The dataset was split into 80% for training and 20% for testing. Voice recordings were segmented into smaller durations (3 to 15 seconds) to improve model robustness, accounting for variable-length input utterances. The AI model was trained for 500 epochs using the loss function described in Sec. 4.3. The Adam optimizer was employed with a learning rate of 3 × 10^−4^, and momentum parameters $\beta_{1}=0.9$ and $\beta_{2}=0.999$. All audio is resampled at 16 kHz.

Our trained model comprises approximately 33.6 million parameters (file size: 128MB), achieves real-time inference on an Intel i7 CPU with an average latency of 73ms, and has a computational cost of 10.2 GMACs.

#### Ethical Considerations.

All user studies conducted in this work follow the ethical standards and guidelines for research involving human participants. The study protocol was reviewed and approved by the Institutional Review Board (IRB) of the authors’ institution. Before participation, all volunteers were informed about the purpose, procedures, and potential risks of the study, and provided their written consent. Participation was entirely voluntary, and participants could withdraw at any time without penalty. During experiments, only audio data relevant to the evaluation of TagMic was collected, and all recorded data were anonymized to protect privacy and confidentiality.

### 5.2 Performance Metrics

We employ both signal-level and perceptual-level metrics to evaluate the performance of voice recovery at the RF reader. The signal-level metrics are objective assessments, while the perceptual-level metrics are subjective evaluations. Specifically, we use the following metrics for evaluation.

- **SI-SDR (signal metric)** represents the Scale-Invariant signal-to-Distortion ratio. It measures the level of signal preservation by calculating the ratio of the target signal to the distortion.
- **LLR (signal-Level metric)** represents the Log-Likelihood ratio. It measures the distortion in speech by comparing the linear predictive coding (LPC) coefficients of the original and processed speech.
- **STOI (perceptual-level metric)** represents the Short-Time objective intelligibility, ranging from 0 to 1. This metric measures the intelligibility of speech by evaluating the similarity between the short-time spectral envelopes of the clean and enhanced signals.
- **PESQ (perceptual-level metric)** is a perceptual evaluation of speech quality, ranging from 1 to 4.5. This metric measures the perceptual quality of speech signals by comparing a reference signal with an enhanced signal.
- **MOS (perceptual-level metric)** represents the Mean Opinion Score. It measures the speech quality based on human ratings, typically ranging from 1 to 5.

For LLR, lower values are preferable. For other metrics, higher scores indicate better-recovered voice quality.

### 5.3 Case Studies

To visually examine the recovered voice signal waveform and better interpret the corresponding metric values, we conducted a set of case studies comparing the recovered voice waveforms against their ground-truth counterparts. In these cases, the recovered voice waveform data was collected while the tag was attached to a person standing approximately 80 cm away from the RF reader.

**Fig. 12.**
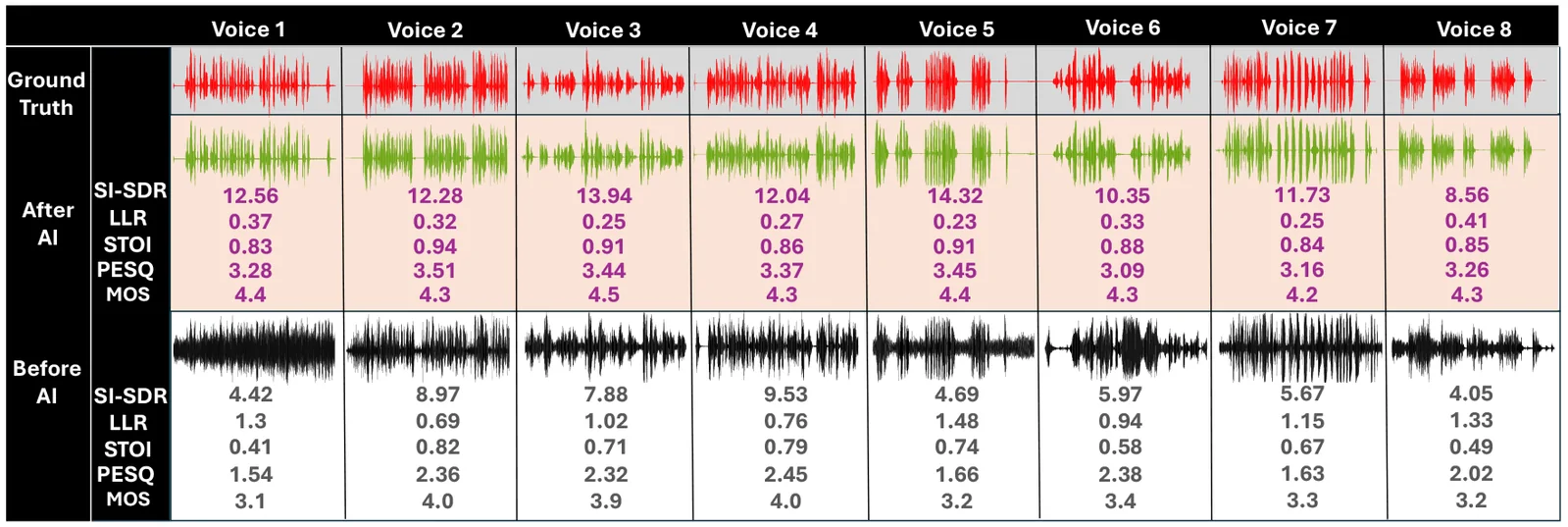
Case studies on the recovered voice signal waveform before and after the AI model as well as their metrics.

Fig. 12 presents our measurement results, showing the voice waveform before and after the AI model alongside the ground-truth voice waveform. The comparisons reveal that the RF reader can recover the voice waveform with high fidelity across all studied cases. Perceptually, one can understand the content of all voice segments almost perfectly. Notably, the AI model enhances the similarity of the recovered waveform to the ground truth. For example, the recovered ‘Voice 1’ waveform before applying the AI model considerably deviates from the original waveform. However, after the process of the AI model, the waveform closely resembles the ground truth, demonstrating the AI model’s effectiveness in voice waveform recovery. Meanwhile, the recovered waveforms for ‘Voice 2,’ ‘Voice 3,’ and ‘Voice 4’ before applying the AI model already exhibit strong similarity to their corresponding ground-truth waveforms. This indicates that, even without the AI model, the proposed signal processing pipeline effectively decodes the voice signal in most cases.

Additionally, Fig. 12 includes the metric values for each recovered voice waveform, both before and after the AI model. These values provide a quantitative understanding of waveform quality, complementing the visual analysis. We hope this combination of visual and numerical data will aid in interpreting the metric values presented in subsequent sections.

### 5.4 Tag Distance

Communication distance is a critical parameter for a wireless microphone. We evaluate the quality of the recovered voice at the RF reader when placing TagMic at different distances. Specifically, TagMic is attached to a participant’s clothing, and the participant speaks at varying distances from the RF reader, ranging from 50 cm to 550 cm in 50 cm increments.

Perceptually, all recovered voice segments remain clearly intelligible. Table 2 presents the quantitative values for those metrics at their 50th percentile. When the distance between TagMic and the RF reader is less than 2 m, the difference in audio quality between the original and recovered voices is perceptually negligible. As the distance increases, TagMic’s performance degrades. However, the AI model’s enhancement becomes more pronounced at greater distances.

It is worth noting that our experimental observations suggest that those metric values do not always correlate directly with human perception. This observation aligns with the findings in prior work [27]: voice signals can remain intelligible even when metric values are low. With the assistance of the AI model, TagMic remains functional and delivers intelligible voice signals even at a distance of 8 m.

### 5.5 Tag Orientation

A key component of TagMic is the parametric resonator (see Fig. 11), designed in a circular shape to efficiently harvest energy from excitation signals. We hypothesize that the energy-harvesting efficiency of TagMic is dependent on its orientation relative to the RF reader. Theoretically, TagMic achieves maximum efficiency when the RF reader is positioned perpendicular to the resonator. To validate this hypothesis, we evaluated TagMic’s performance across various orientations (0° to 180° in 30° increments) and distances (1 m to 5 m in 1 m increments).

**Fig. 13.**
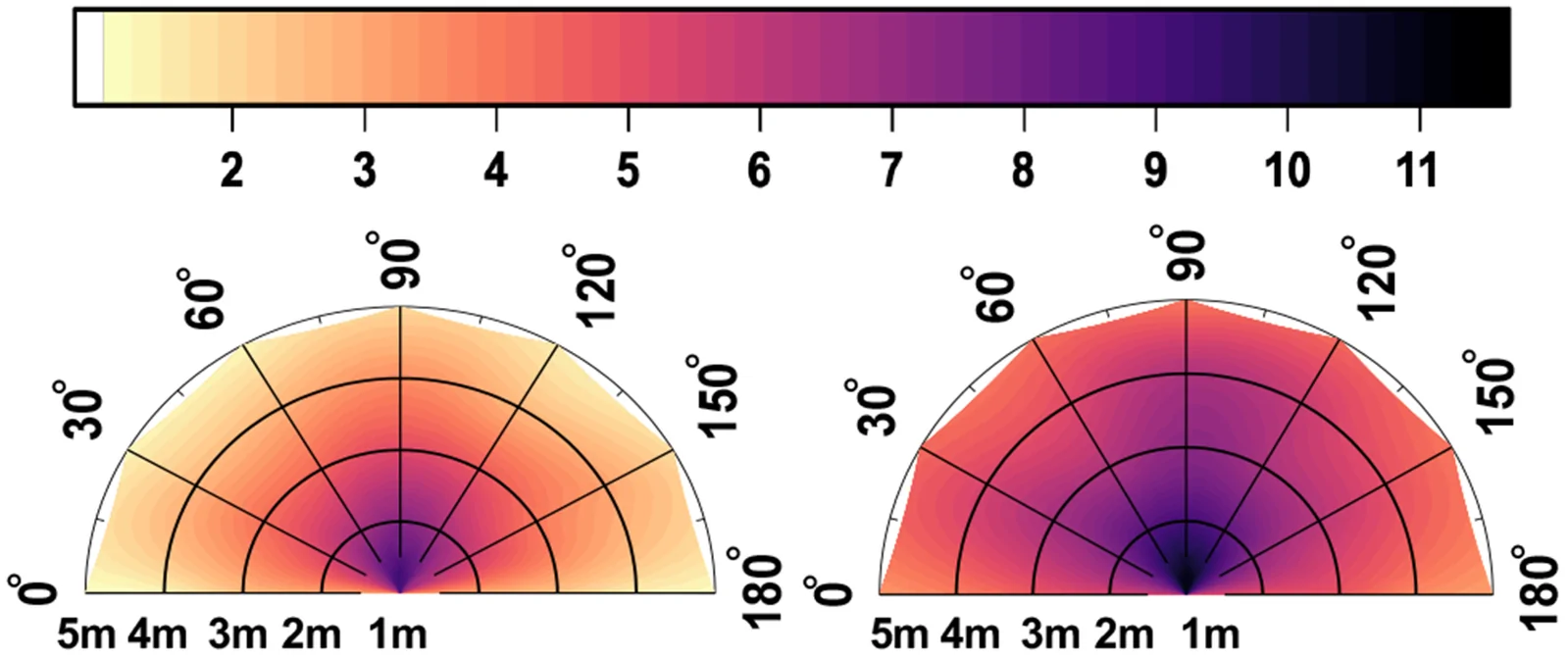
Measured SI-SDR when TagMic Orientations

In all cases, the recovered voice at the RF reader was intelligible. Fig. 13 shows the SI-SDR results measured at the RF reader. The left panel illustrates the SI-SDR of the recovered voice signal without AI enhancement, while the right panel depicts the SI-SDR after AI enhancement. The results indicate that the SI-SDR performance of TagMic is primarily influenced by the distance to the RF reader, with orientation playing a less significant role than expected. This could be attributed to the rich scattering properties of indoor environments, which help maintain consistent energy harvesting and voice recovery performance regardless of orientation.

### 5.6 Resilience to Blockage

**Fig. 14.**
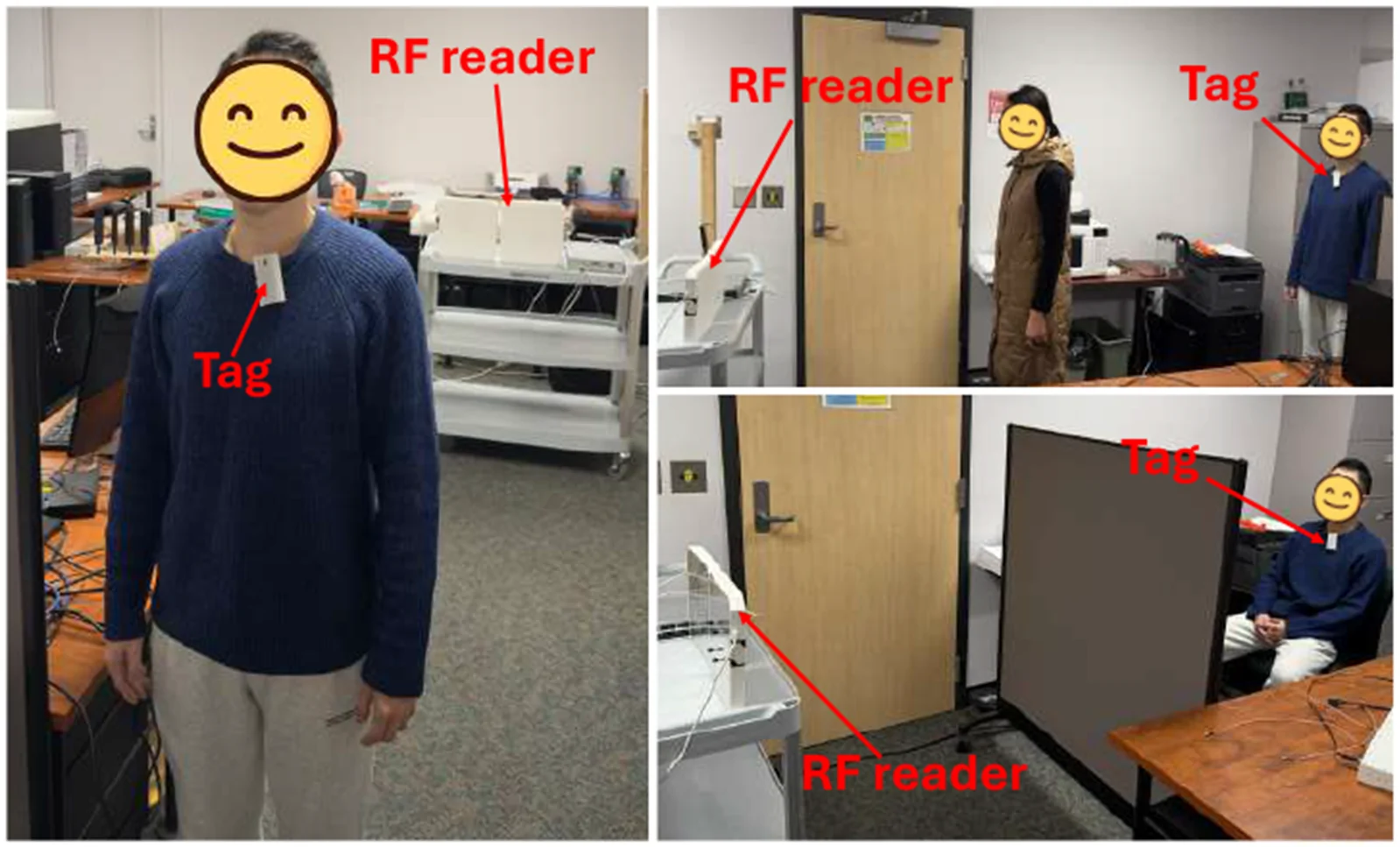
Blockage study.

Blockage is an important factor in indoor environments. To evaluate TagMic’s resilience to blockages, we conducted experiments to evaluate the performance of TagMic in three typical scenarios: (i) the speaker faces away from the RF reader (see the left image in Fig. 14); (ii) a person obstructs the line-of-sight path between TagMic and the RF reader (see the top-right image in Fig. 14); and (iii) a piece of furniture blocks the line-of-sight path between TagMic and the RF reader (see the bottom-right image in Fig. 14). These scenarios are referred to as ‘Blockage 1,’ ‘Blockage 2,’ and ‘Blockage 3,’ respectively. In all cases, the distance between TagMic and the RF reader was approximately 3 meters.

**Fig. 15.**
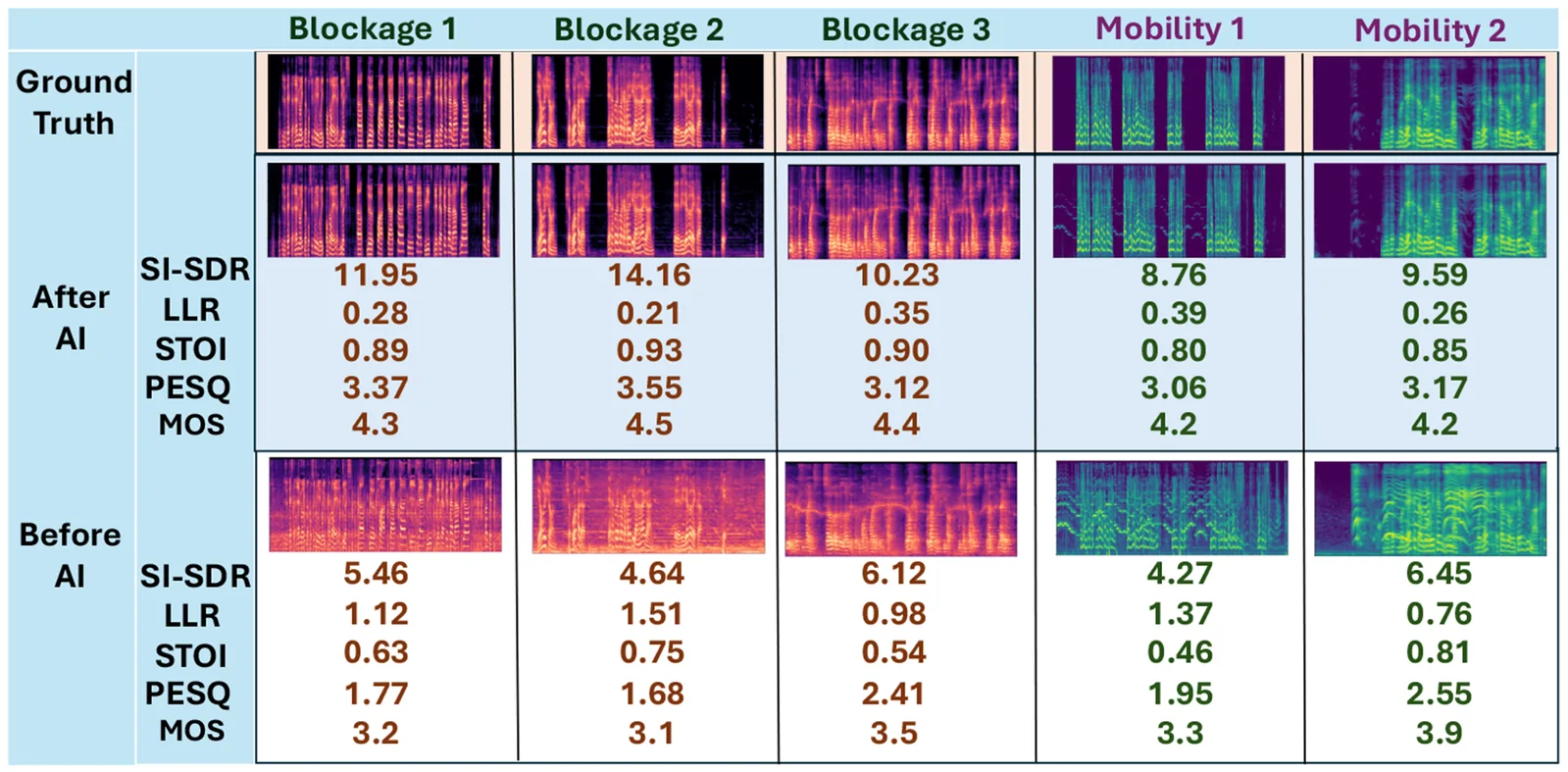
Mel spectrogram of the recovered and original voices in the presence of blockage and mobility.

Fig. 15 presents the experimental results as mel spectrograms (a standard method for sound analysis) along with the corresponding metric values. The findings demonstrate that, with the AI model, TagMic effectively reconstructs voice signals with high similarity to the ground-truth voice. Notably, TagMic Exhibits strong resilience to all three blockage scenarios. Specifically, in most cases involving furniture, TagMic can recover intelligible voice signals even without the AI model.

### 5.7 Mobility

**Fig. 16.**
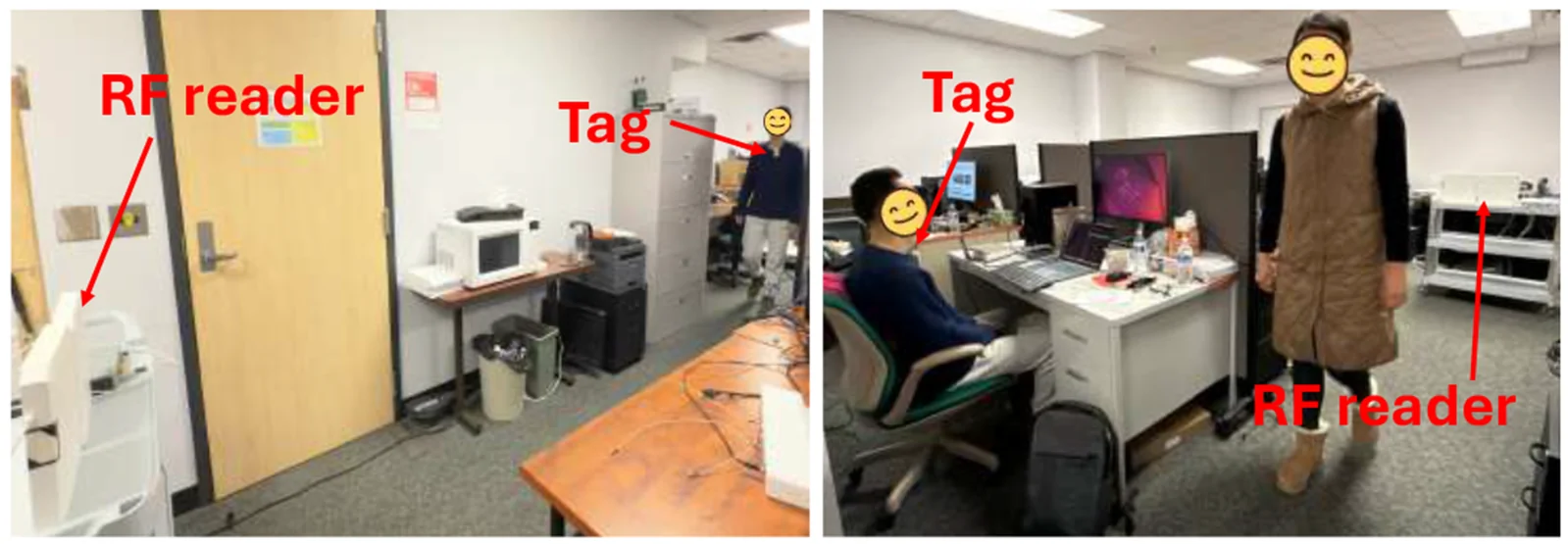
Mobility study

A wireless microphone must support mobility for practical use. To validate the usability of TagMic, we conducted mobility tests in two cases: (i) the speaker walks in front of the RF reader, with the distance varying from 2.5 m to 5.0 m (see the top image in Fig. 16); and (ii) the speaker remains seated on a chair while an interfering person moves around the speaker and the RF reader (see the bottom image in Fig. 16). These scenarios are referred to as ‘Mobility 1’ and ‘Mobility 2,’ respectively. Fig. 15 presents our experimental results. The findings indicate that TagMic’s performance degrades slightly when the speaker is in motion. However, TagMic consistently maintains satisfactory performance in both scenarios, with all recovered voices being of high quality.

### 5.8 Multi-user Support

**Fig. 17.**
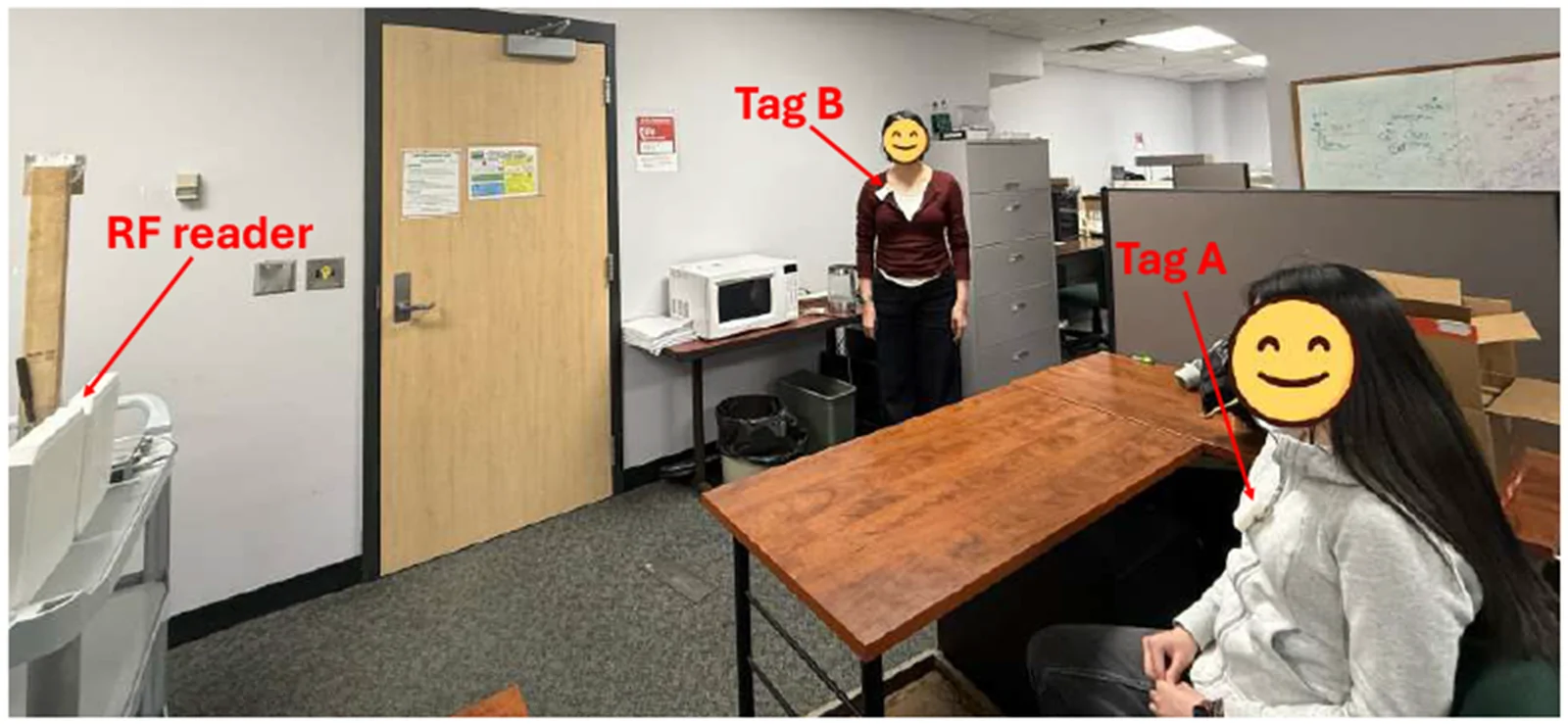
Multi-user support.

**Fig. 18.**
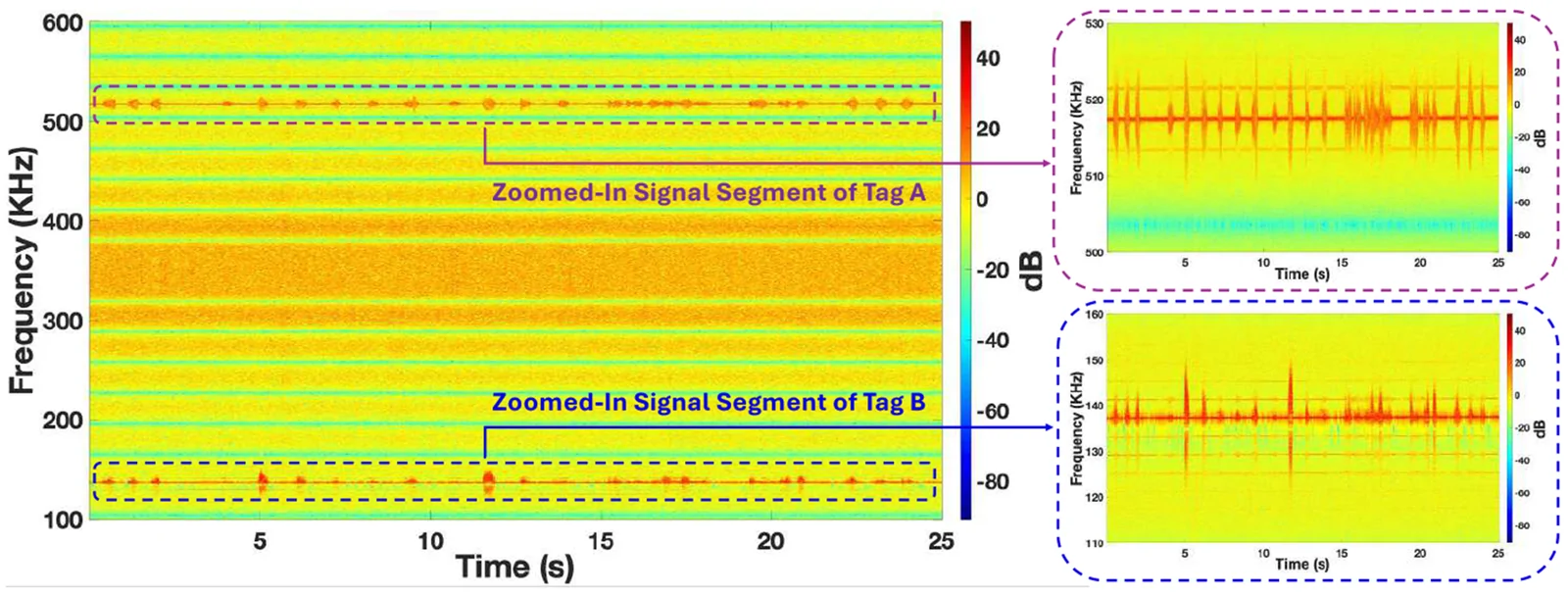
Spectrogram of two RF tags’ reflection signal.

TagMic can support multi-user operation through frequency division. By adjusting the number of coil windings, the ’circular-mode’ resonance frequency ($f_{c}$) of TagMic can be tuned to a unique operation frequency. To validate this feature, we fabricated a second TagMic tag that has a different number of coil windings from the first tag. Fig. 17 illustrates the multi-user testing scenario, where two participants wear Tag A and Tag B, respectively. Fig. 18 presents the spectrogram captured when both tags were concurrently active and modulated by voice signals. The spectrogram shows two separate clusters of frequency-modulated carriers, each from one tag, centered around distinct frequencies. In this experiment, the frequency separation was approximately 380 kHz. This frequency separation is sufficiently large to prevent mutual interference, allowing the RF reader to isolate and demodulate each voice stream. After AI enhancement, the demodulated voice streams from Tag A and Tag B achieved quality comparable to single-tag operation, with SI-SDR values of 11.75 and 12.37, LLR values of 0.29 and 0.23, STOI values of 0.91 and 0.93, PESQ values of 3.35 and 3.46, and MOS values of 4.1 and 4.3, respectively. More importantly, our experimental results demonstrate that this frequency separation remains stable regardless of the relative distance between the tags, their individual orientations, or their operational status (stationary or mobile). This ensures reliable signal separation under diverse real-world conditions.

### 5.9 Overall Performance

We conducted extensive experiments to evaluate TagMic’s performance across a wide range of scenarios, considering factors such as varying tag distances, orientations, mobility speeds, and voice source types. Our objective was to comprehensively assess its effectiveness and reliability under real-world conditions. A total of 1500 minutes of voice data were collected for this evaluation.

**Fig. 19.**
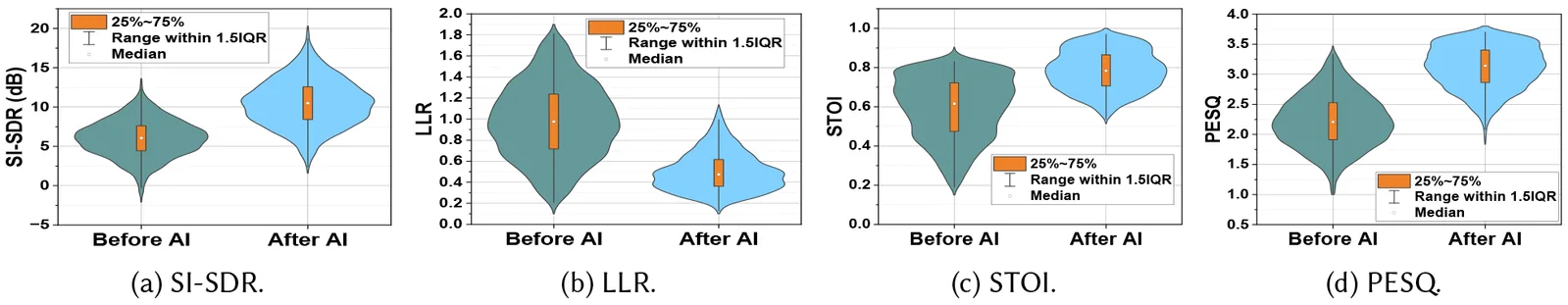
Overall performance of TagMic in various conditions.

Twelve volunteers participated in a perceptual evaluation, classifying the recovered voices into three categories: low quality, medium quality, and high quality. Approximately 20% of the voices were classified as low quality, 45% as medium quality, and 35% as high quality. In addition to this subjective evaluation, Fig. 19 presents a statistical summary of the metric values across all scenarios. The results demonstrate that the AI model significantly enhances the overall voice quality, ensuring that the majority of the voices are intelligible after processing.

## 6 RELATED WORK

### 6.1 Batteryless Microphones Using RF Backscatter

RF backscatter offers a compelling pathway for ultra-low-power communication by reflecting ambient RF signals. In general, two types of RF backscatter systems have been explored to realize batteryless data communications.

- *Digital Backscatter Microphones:* Platforms like the Wireless Identification and Sensing Platform (WISP) [17] and networked systems like MultiScatter [14] can be utilized to implement a batteryless wireless microphone. They digitize the audio signal before modulating it onto the backscattered carrier frequency, offering programmability and compatibility with standards like EPC Gen2 [17]. However, the energy required for signal digitization and digital data processing limits its operation in a continuous fashion due to their reliance on energy harvesting circuits. As a result, these approaches are limited to a low-duty-cycle operation and cannot support continuous voice streaming [14, 17, 34].
- *Analog Backscatter Microphones:* To overcome the power limitations of digital components, researchers have developed purely analog systems. Early concepts like the Great Seal Bug [29] demonstrated direct modulation of RF carriers via physical vibrations. Three modulations have been exploited for this purpose. The first one is Frequency Modulation (FM). For example, RF Bandaid [26] directly maps sensor outputs to frequency changes, achieving FM without digital conversion. MARS [1] advances this, operating below 1 μW using a modified Clapp oscillator. The second one is Amplitude Modulation (AM) and Impedance Modulation. For instance, the Battery-Free Phone [34] modulated a JFET’s impedance using an electret microphone’s output. The third one is Advanced Modulation and Arrays. For example, MicArray [53] employed Pulse Position Modulation (PPM) and Differential PPM (DPPM) for synchronous multi-track audio recording.

While these works demonstrate important design insights, they are constrained by tradeoffs that limit their suitability for continuous, high-quality voice streaming. Specifically, Battery-Free Phone [34] employs a hybrid analog-digital design involving a microcontroller, impedance modulation, and energy storage, which necessitates duty cycling and limits communication continuity. In contrast, TagMic eliminates all active electronics and digital control logic, relying entirely on passive components for continuous, real-time analog frequency modulation. Its dual-resonator design separates the excitation and reflection frequencies, fundamentally resolving self-interference and enabling reliable multi-tag operation. MARS [1] is designed for general-purpose low-power sensing (e.g., touch, swipe, and short speech bursts), but its minimalist design sacrifices signal fidelity and consistency. TagMic, by comparison, achieves continuous analog modulation with enhanced resilience through its multi-carrier architecture and precise separation of oscillation frequencies. It also avoids complex components like zero-threshold MOSFETs used in MARS. MicArray [53] targets batteryless operation but depends on external power for FPGA-based digital signal processing. It also relies on time-multiplexed synchronization and digital pulse modulation schemes, introducing hardware complexity and requiring coordinated multi-channel sampling. TagMic instead uses a fully analog architecture with passive frequency modulation, removing the need for synchronization or digital logic and supporting scalable deployment in multi-user environments.

Despite the unique design tradeoffs of these systems, they all share a fundamental limitation: the self-interference challenge inherent in conventional backscatter, where the tag’s reflective signal overlaps with the reader’s excitation frequency [1, 13, 14, 17, 26, 28, 34, 35, 37, 53]. This overlap arises because the excitation signal is orders of magnitude stronger than the weak backscattered reflection, significantly impairing signal demodulation. Although harmonic backscatter [18, 48] offers one solution by reflecting at integer multiples of the excitation frequency, this approach typically requires higher activation power for the nonlinear components, making it difficult to directly modulate analog voice signals.

Table 1 provides a comparative summary of prior systems, highlighting TagMic’s distinctive integration of frequency separation, analog modulation, and support for continuous, battery-free operation.

### 6.2 Non-backscatter RF-based Sound Sensing

In addition to RF backscatter techniques, other RF techniques have been studied to detect sound or voice from human. One popular approach is radar-based sound Sensing. Systems like RadioMic [20, 21], Radio2Speech [54], mmspy [3], mmecho [11], RFMic-Phone [32], and others [7-9, 38-40, 43-45, 49] use mmWave radar to detect minute vibrations caused by sound waves. They can offer capabilities like through-wall sensing [41] and noise resilience [23, 25], sometimes combining radar with traditional microphones [32]. However, these sensing methods typically require significantly more complex hardware and higher power consumption compared to RF backscatter tags [5, 20-23, 50-52, 54]. Other than FMCW mmWave radar, UWB radar is another sensor used for sound sensing. Prior work like UWHear [42] use UWB radar for audio separation, while RADIOSES [24] combines audio and RF modalities to improve speech separation quality. These demonstrate sophisticated capabilities but also entail system complexity and energy demands. While these alternative RF methods offer unique advantages (e.g., privacy, environmental robustness), TagMic differs from them by providing a reliable, continuous voice streaming solution. This is because this radar-based approach can only work in a controllable setting where the sound source is static and its location is known a priori. In contrast, TagMic can work in a generic setting and for mobile voice sources.

## 7 CONCLUDING REMARKS

In this paper, we introduced a batteryless wireless microphone through the meticulous design of a backscatter tag. Unlike traditional backscatter tags, our tag reflects radio signals at a frequency different from its excitation signal, effectively addressing the notorious self-interference issue in conventional RF backscatter tags. By adopting a dual-resonator structure, the tag modulates the voice signal onto its resonance frequency, eliminating the need for digitalization and creating an energy-efficient voice FM system. We have fabricated TagMic and evaluated its performance in realistic scenarios. Experimental results confirm its practicality and efficiency under various conditions.

While TagMic has demonstrated significant potential and reliable performance, it has several limitations that warrant discussion: *(i) Dependence on reader infrastructure*. TagMic requires an excitation signal from a dedicated RF reader. This dependency on custom infrastructure may limit plug-and-play deployment in environments that lack compatible RF readers or regulated RF spectrum access. *(ii) Limited communication range compared to active systems*. Although our system achieves up to 8 meters of communication distance, this range is shorter than conventional battery-powered wireless microphones, which can operate over tens of meters with actively transmitted signals. The reliance on passive backscatter inherently limits the available signal power and thus the effective range. *(iii) Scalability with large numbers of tags remains unexplored*. While our system supports two concurrent tags through frequency division, scalability to tens or hundreds of users has not been studied. In dense acoustic scenarios, ensuring sufficient spectral separation and mitigating inter-tag interference may present additional challenges, particularly in mobile or ad-hoc deployments. We envision that some of these limitations can be addressed through future advances in low-power RF front-end design, integrated reader hardware, and adaptive signal processing pipelines. Overall, TagMic should be regarded as a lab-validated prototype rather than a ready-to-deploy commercial system. While our experiments demonstrate the feasibility of continuous, battery-free voice streaming, the current design has been evaluated under controlled laboratory conditions with research-grade hardware. Additional engineering efforts, such as developing dedicated low-cost RF readers, optimizing the hardware form factor, and compressing the AI model for edge deployment, will be necessary before TagMic can transition from a prototype to practical real-world applications.

## Figures and Tables

**Table 1.** Comparison of RF Backscatter-based Microphone.

Feature	Continuous Voice Streaming?	Self- Interference	Batteryless?	Multiple Tags?	Circuit Type	Modulation	Communication Range
Hybrid A/D [35]	No	Yes	Yes	No	Analog + Digital	ASK/PSK + AM	2.7 m
Battery Free Phone [34]	No	Yes	Yes	No	Analog + Digital	OOK/ASK + AM	9.4 m
RF Bandaid [26]	No	Yes	Yes	No	Analog	FM	4 m
Multi Scatter [14]	No	Yes	Yes	Yes	Digital	FSK + ASK	45.7 m
MARS [1]	No	Yes	Yes	Yes	Analog	FM	9 m
MicArray [53]^†^	Yes	Yes	No	No	Analog + Digital	PM	28 m
WISP 6.0 [17]	No	Yes	Yes	No	Analog + Digital	ASK/PSK + FM0	1.5 m
**TagMic (This work)**	**Yes**	**No**	**Yes**	**Yes**	**Analog**	**FM**	**8 m**

† MicArray targets batteryless operation, but its implementation relies on external power for FPGA-based digital processing on the backscatter tag.

**Table 2.** TagMic’s performance at different distances.

	SI-SDR^↑^		LLR^↓^		STOI^↑^		PESQ^↑^		MOS^↑^	
	w/o AI	w/ AI	w/o AI	w/ AI	w/o AI	w/ AI	w/o AI	w/ AI	w/o AI	w/ AI
50 cm	12.1	14.65	0.25	0.21	0.82	0.97	3.21	3.65	4.5	4.5
100 cm	9.95	11.33	0.27	0.24	0.77	0.91	3.04	3.57	4.3	4.5
150 cm	8.90	9.81	0.37	0.3	0.75	0.82	2.98	3.20	4.0	4.2
200 cm	7.15	8.57	0.49	0.41	0.69	0.76	2.85	2.93	3.5	4.0
250 cm	5.64	7.42	0.65	0.52	0.61	0.73	2.71	2.88	3.0	3.5
300 cm	4.97	7.05	0.71	0.53	0.55	0.72	2.49	2.73	3.0	3.5
350 cm	4.05	6.86	0.82	0.58	0.52	0.67	2.33	2.61	2.7	3.5
400 cm	3.16	6.11	0.86	0.61	0.40	0.65	1.95	2.55	2.5	3.35
450 cm	2.45	4.63	0.99	0.77	0.38	0.6	1.78	2.17	2.0	3.2
500 cm	2.05	3.56	1.10	0.89	0.35	0.56	1.66	2.01	2.0	3.1
550 cm	1.77	3.27	1.35	0.97	0.30	0.55	1.42	1.93	1.5	2.85

## CCS Concepts:

• Human-centered computing → Ubiquitous and mobile computing systems and tools; • Hardware → Sensor devices and platforms.
